# Antibiotic Resistance Mediated by the MacB ABC Transporter Family: A Structural and Functional Perspective

**DOI:** 10.3389/fmicb.2018.00950

**Published:** 2018-05-28

**Authors:** Nicholas P. Greene, Elise Kaplan, Allister Crow, Vassilis Koronakis

**Affiliations:** ^1^Department of Pathology, University of Cambridge, Cambridge, United Kingdom; ^2^School of Life Sciences, University of Warwick, Coventry, United Kingdom

**Keywords:** antibiotic resistance, tripartite efflux pump, MacB, mechanotransmission, ABC transporter, lantibiotic, membrane protein, antimicrobial resistance

## Abstract

The MacB ABC transporter forms a tripartite efflux pump with the MacA adaptor protein and TolC outer membrane exit duct to expel antibiotics and export virulence factors from Gram-negative bacteria. Here, we review recent structural and functional data on MacB and its homologs. MacB has a fold that is distinct from other structurally characterized ABC transporters and uses a unique molecular mechanism termed mechanotransmission. Unlike other bacterial ABC transporters, MacB does not transport substrates across the inner membrane in which it is based, but instead couples cytoplasmic ATP hydrolysis with transmembrane conformational changes that are used to perform work in the extra-cytoplasmic space. In the MacAB-TolC tripartite pump, mechanotransmission drives efflux of antibiotics and export of a protein toxin from the periplasmic space via the TolC exit duct. Homologous tripartite systems from pathogenic bacteria similarly export protein-like signaling molecules, virulence factors and siderophores. In addition, many MacB-like ABC transporters do not form tripartite pumps, but instead operate in diverse cellular processes including antibiotic sensing, cell division and lipoprotein trafficking.

## Introduction

**Figure 1:**
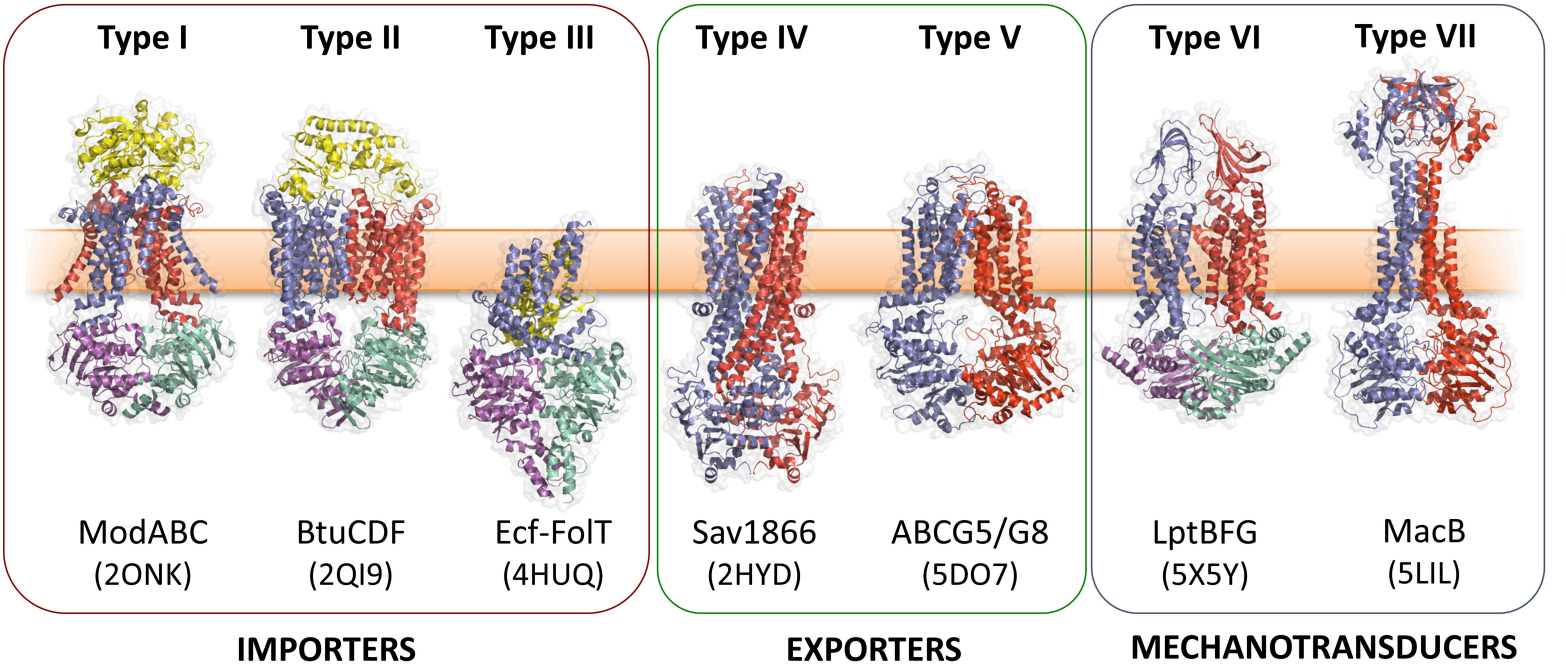
Seven ABC transporter superfamilies. A single representative from each superfamily is shown colored by protein chain. PDB identifiers are given in parentheses. The seven ABC folds are here further divided into three “classes” based on function. Families I-III are importers, Type IV and V are exporters and Types VI and VII are mechanotransducers. Note that the two ABC exporter families here termed Type IV and Type V are also sometimes referred to as type I and II ABC exporters. From left to right: the molybdate transporter (Hollenstein et al., 2007) (ModABC), vitamin B12 transporter (Korkhov et al., 2012) (BtuCDF), folate importer (Xu et al., 2013) (Ecf-FolT), multidrug exporter (Dawson and Locher, 2006) (Sav1866), the sterol transporter (Lee et al., 2016) (ABCG5/G8), the lipopolysaccharide extractor (Luo et al., 2017) (LptBFG) and the enterotoxin and macrolide transporter (Crow et al., 2017) (MacB). Folds are named by extension of a previously established convention (ter Beek et al., 2014). Adapted from Crow et al. (2017).

ABC (ATP-binding cassette) transporters are present in all three domains of life, and mediate transmembrane transport of a diverse array of substrates including drugs, sugars, ions, amino acids and proteins (ter Beek et al., 2014; Locher, 2016). All ABC transporters possess conserved nucleotide binding domains (NBDs) that facilitate power generation through ATP hydrolysis, and transmembrane domains (TMDs) that determine transporter function. The NBDs at the core of all ABC transporters are homologous, while the TMDs are structurally heterogeneous and proposed to have discrete evolutionary origins (Wang et al., 2009; ter Beek et al., 2014; Locher, 2016). Analysis of available ABC transporter crystal structures shows that there are at least seven structurally distinct ABC transporter folds, of which MacB is the most-recently determined representative (Figure 1). MacB is therefore the “holotype” for the Type VII ABC transporter superfamily (Crow et al., 2017), and the first structurally-characterized member of a clade of ABC transporters classified by Saier as the ABC3 superfamily (Wang et al., 2009).

ABC transporters with roles in antibiotic resistance are typically exporters, and operate by some variation of an alternating access mechanism (see below) (ter Beek et al., 2014; Locher, 2016). In bacteria, ABC exporters possess six transmembrane helices (TMHs) and operate as dimers. In eukaryotes, they typically exist as apparent fusions of two half transporters, that are otherwise structurally similar to their bacterial counterparts (Locher, 2016). Key examples of ABC exporters include Sav1866 from *Staphylococcus aureus* (Dawson and Locher, 2006), *Bacillus subtilis* LmrA (van Veen et al., 1996) and P-glycoprotein from humans (Aller et al., 2009).

Structures of ABC transporters in the presence and absence of ATP reveal different conformations which led to the proposal of an alternating access mechanism for transport (ter Beek et al., 2014; Locher, 2016). ATP-dependent conformational changes in the NBDs cause the TMDs to cycle between “inward open” and “outward open” states allowing substrate to be bound at one side of the membrane and released on the other. In the inward open state, the NBDs are parted and substrates can bind to a site at the interface of the TMDs, exposed to the cytoplasmic side of the membrane (Johnson and Chen, 2017). ATP binding promotes tight association of the NBDs and is communicated to the TMDs through a conserved coupling helix (Dawson and Locher, 2006). The resultant reorganization of the TMD results in an “outward-open” state with a reduced affinity for substrate allowing release on the distal side of the membrane (Ramachandra et al., 1998; Johnson and Chen, 2018). ATP hydrolysis then resets the transporter to an inward facing conformation. The stoichiometry of ATP hydrolysis per translocation event is unclear since heterodimeric transporters with only one functional NBD are translocation competent (Zutz et al., 2011). Transport may be further aided by the transmembrane proton electrochemical gradient (Singh et al., 2016). Structures of occluded states, in which the binding site is not accessible to either side of the membrane, lack substrate but represent plausible intermediates on the pathway between inward and outward open states (Choudhury et al., 2014; Lin et al., 2015a; Bountra et al., 2017). Variations of the mechanism in which only the outward facing state takes part in transport have been proposed (Perez et al., 2015; Locher, 2016). Indeed, it has been suggested that the diverse structures and substrates of ABC transporters are incompatible with a single unified transport mechanism (Locher, 2016).

In Gram-positive bacteria, ABC transporters are widely used to expel xenobiotics (Lubelski et al., 2007). Antibiotic efflux by Gram-negative ABC transporters has been less well studied although expression of *Stenotrophomonas maltophila* SmrA (Al-Hamad et al., 2009) or *Serratia marcescens* SmdAB (Matsuo et al., 2008), in hypersusceptible *E. coli*, provides resistance to multiple drugs including norfloxacin and tetracycline. Overexpression of *E. coli* MsbA confers resistance to multiple drugs in *E. coli* and *Lactococcus lactis* (Reuter et al., 2003; Woebking et al., 2005).

Most ABC exporters operate independently to transport substrates across the cytoplasmic membrane in which they are embedded. However, in Gram-negative bacteria specific ABC transporters can form part of tripartite efflux pumps, larger assemblies that span the entire cell envelope and mediate transport across the outer membrane.

## Tripartite efflux pumps (TEPs): bacterial nanomachines driving antibiotic efflux

**Figure 2:**
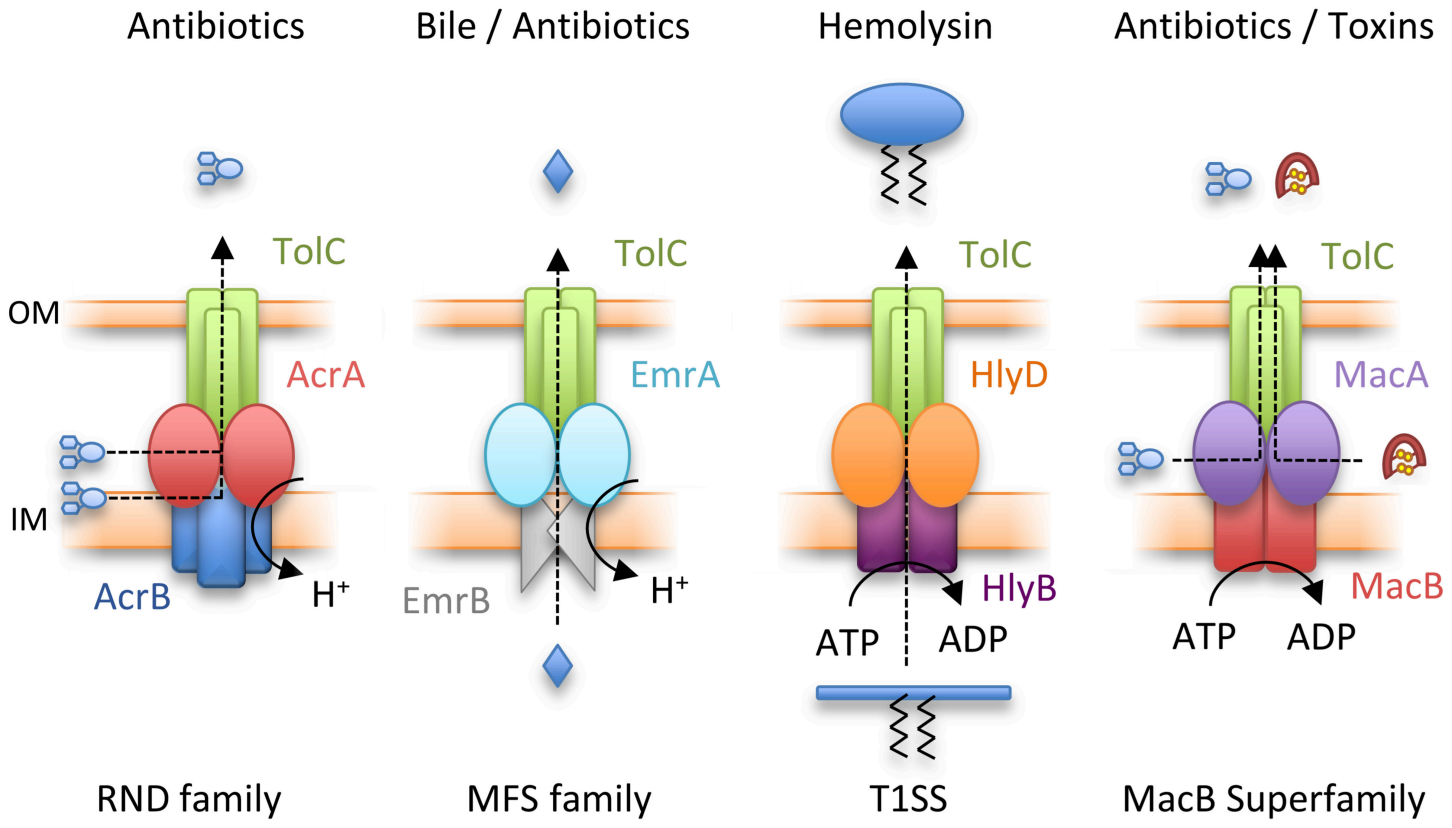
Four classes of Tripartite Efflux Pump. From left to right, Resistance-Nodulation-Cell Division (RND) family pump, AcrAB-TolC; Major Facilitator Superfamily (MFS) pump, EmrAB-TolC; Type I secretion system (T1SS), HlyBD-TolC; MacB superfamily pump, MacAB-TolC. IM and OM indicate the inner and outer membranes respectively.

Gram-negative bacteria use tripartite efflux pumps that span both inner and outer membranes to export and efflux noxious molecules including antibiotics. They are a major determinant of multidrug resistance (Hinchliffe et al., 2013). The pumps consist of an outer membrane exit duct exemplified by *E. coli* TolC (Koronakis et al., 2000), a periplasmic adaptor protein and an inner membrane transporter. The periplasmic adaptors have a conserved, multi-domain, architecture consisting of membrane proximal (MP), β-barrel, lipoyl and hairpin domains, although some adaptors lack one of these domains (Greene et al., 2013; Hinchliffe et al., 2014). The energy-transducing inner membrane transporter comes from one of four distinct classes. The Resistance-Nodulation-Cell Division (RND) e.g., AcrB and Major Facilitator Superfamily (MFS) transporters e.g., EmrB both couple export to dissipation of the transmembrane electrochemical ion gradient. Conversely, ATP hydrolysis is used by two distinct ABC transporters, HlyB and MacB, that account for the third and fourth TEP types (Figure 2). In *E. coli*, all four TEP classes use one outer membrane efflux protein, TolC (Hinchliffe et al., 2013).

### The different classes of transporter powering TEPs

Structures of the best characterized RND inner membrane component, AcrB, were first determined over 15 years ago. AcrB functions as a homotrimer, with each monomer containing 12 TMHs and an extensive periplasmic domain projecting 70 Å into the periplasm (Murakami et al., 2002). Subsequent structures revealed asymmetric AcrB trimers in which individual AcrB monomers adopted different conformational states (Murakami et al., 2006; Seeger et al., 2006). This led to the proposal of a transport mechanism in which each monomer cycles between open, loose and tight substrate-binding states to effect transport. The broad substrate specificity of AcrB is explained by multiple binding sites for drugs and entry routes from both the outer leaflet of the inner membrane and the periplasm (Nakashima et al., 2011; Eicher et al., 2012; Zwama et al., 2018). More recently, structures of the assembled AcrAB-TolC complex have been obtained using negative-stain and cryo-electron microscopy (cryo-EM) (Du et al., 2014; Daury et al., 2016; Wang et al., 2017).

MFS transporters associating with TEPs have been less well studied. In *E. coli*, the exemplar is EmrB which, with its cognate adaptor EmrA, confers resistance to hydrophobic compounds including the proton-motive force uncoupler, CCCP, and nalidixic acid (Lomovskaya and Lewis, 1992). EmrB is predicted to have 14 TMHs and, unlike other TEP transporters, lacks a substantial periplasmic domain (Tanabe et al., 2009). Consistent with this, EmrA does not have an MP domain (Hinchliffe et al., 2014) that in other transporters forms extensive interactions with the transporter periplasmic domain (Du et al., 2014). MFS transporters have been reported to operate either as monomers or dimers (Quistgaard et al., 2016). Purified EmrB was reported to form dimers (Tanabe et al., 2009) whereas the diameter of a modeled EmrA hexamer was compatible with monomeric EmrB (Hinchliffe et al., 2014). Further studies are required to understand the EmrB stoichiometry *in vivo* and the EmrA-EmrB interactions underpinning pump assembly.

In *E. coli*, two very different ABC transporters form TolC-dependent TEPs; HlyB and MacB. HlyB acts in concert with the adaptor HlyD to export the large protein toxin, hemolysin A (HlyA), from the cytoplasm across both membranes in a concerted step (Thanabalu et al., 1998). Topological analysis, and the crystal structure of a peptide exporting homolog (PCAT1), demonstrate HlyB has a classic 6-TMH topology with the NBD fused to the C-terminus (Lin et al., 2015a). Conversely, sequence analysis of *E. coli* MacB suggested that it has an atypical topology. An N-terminal cytoplasmic NBD is followed by four TMHs, with a large periplasmic domain of approximately 200 amino acids situated between TMH1 and TMH2. The 4-TMH topology predicted from amino acid sequence was experimentally confirmed by site-specific chemical modification of single cysteine residues (Kobayashi et al., 2003). Subsequent bioinformatic analysis revealed the MacB architecture is widespread throughout bacterial genomes (Khwaja et al., 2005; Wang et al., 2009).

### The MacAB-TolC TEP mediates antibiotic resistance and export of virulence factors

MacB, along with its periplasmic adaptor protein MacA, was first identified in a screen of *E. coli* transporter genes as providing resistance to macrolide drugs in a strain lacking the major RND efflux pump AcrAB (Kobayashi et al., 2001). Further studies demonstrated that expression of MacAB increases *E. coli* resistance to colistin and bacitracin (Crow et al., 2017). Additionally, the MacAB-TolC TEP supports the export of small proteins such as enterotoxin STII (Yamanaka et al., 2008), and is suggested to export the heme-precursor protoporphyrin (Turlin et al., 2014).

The role of the MacAB-TolC TEP has been investigated in other Gram-negative species. *S. maltophila* MacAB confers resistance to a variety of macrolides, aminoglycosides and polymyxins (Lin et al., 2014). In *Neisseria gonorrhoeae*, mutations in the *macAB* promoter increase macrolide resistance in a strain lacking the RND pump MtrCDE (Rouquette-Loughlin et al., 2005). *Acinetobacter baumannii* MacAB expression is significantly upregulated in both multidrug-resistant clinical strains (Lin et al., 2015b), and colistin-resistant strains devoid of LPS (Henry et al., 2012). Expression of the *Vibrio cholerae* MacAB homolog VarDEF increases resistance to four different macrolides by 8-fold or greater (Lin et al., 2017), and these genes were upregulated in presence of polymyxin B (Matson et al., 2017). In *Pseudomonas* species, homologs of MacB secrete toxins (Balibar et al., 2005; Dubern et al., 2008; Lim et al., 2009; Cho and Kang, 2012; Li et al., 2013), and siderophores such as pyoverdine (Imperi et al., 2009; Hannauer et al., 2010). Deletion of MacB impacts virulence of *Salmonella* in a mouse model (Nishino et al., 2006), possibly by promoting survival within macrophages (Bogomolnaya et al., 2013). Taken together, MacB, in concert with the adaptor MacA and the outer membrane exit duct, TolC, can underpin efflux of a variety of drugs and export of virulence factors from multiple Gram-negative bacterial species. While TolC expression is constitutive, expression of MacAB in *Salmonella* and *E. coli* is regulated by the PhoPQ two-component system (Nishino et al., 2006). Among other stimuli, PhoPQ senses and responds to host antimicrobial peptides and peptide antibiotics (Bader et al., 2005; Prost et al., 2007). Thus, MacAB production is likely to be induced in response to challenge with these agents.

## Structural biology of the MacAB-TolC system

**Figure 3:**
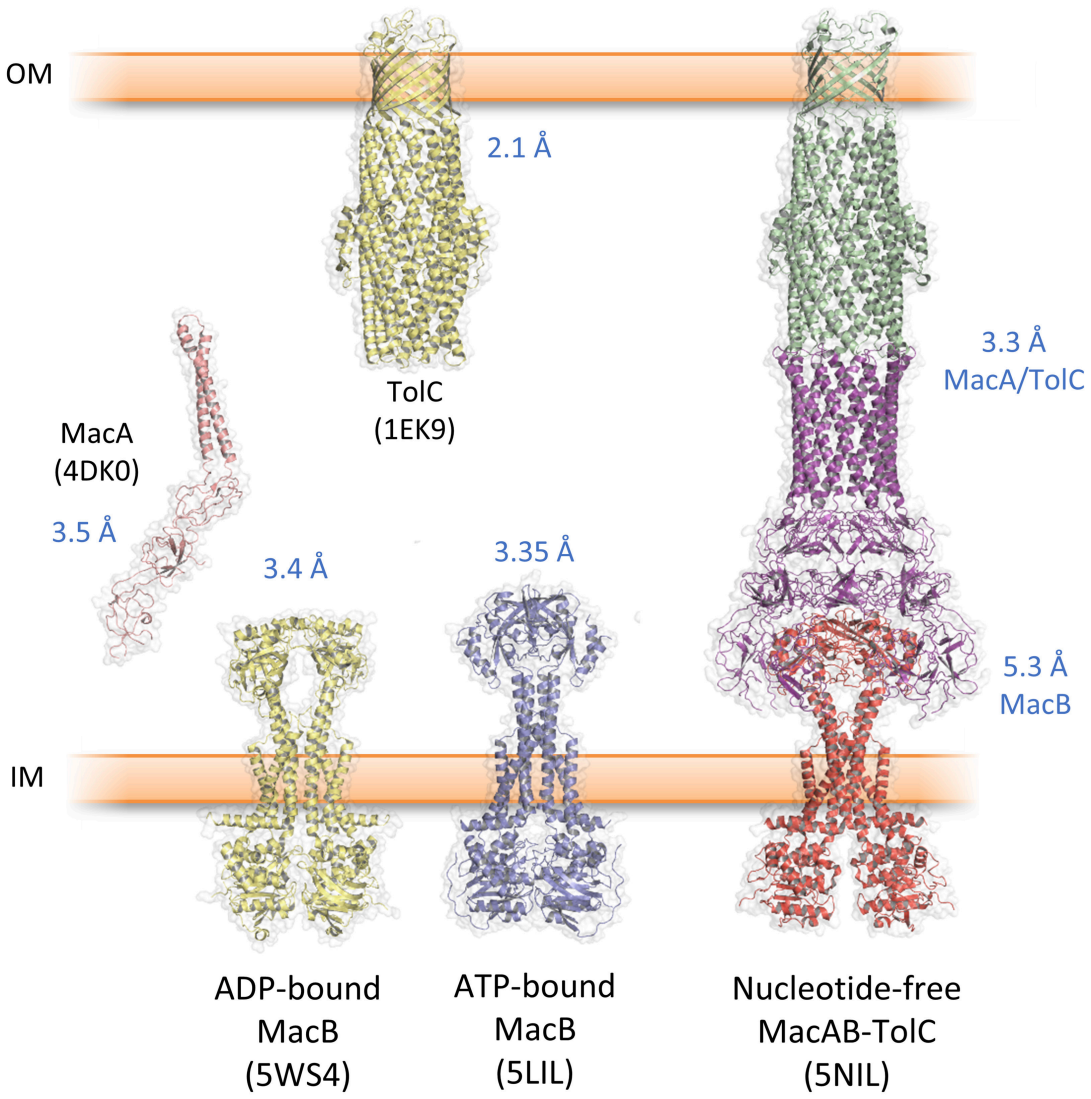
Key structures from the MacAB-TolC tripartite efflux pump. Protein data bank accession codes are given in brackets and the reported resolution indicated in blue text. The complete MacAB-TolC pump is a cryo-EM structure with higher resolution in the MacA/TolC region and lower resolution in MacB (Fitzpatrick et al., 2017).

Studies by the Zgurskaya lab demonstrate that the ATPase activity of reconstituted MacB is dependent on intact MacA. Furthermore, MacAB mediated antibiotic resistance *in vivo* requires the presence of the outer membrane efflux channel TolC (Tikhonova et al., 2007). Taken together, these data confirm that MacAB-TolC forms a functional TEP. Recently, crystal structures of the individual components, and a cryo-EM structure of the entire pump, have been elucidated, providing substantive insight into the assembly and function of the MacB-powered TEP (Table 1, Figure 3).

**Table 1:** Structures in the Protein Data Bank associated with MacAB-TolC.

**Protein**	**Organism**	**PDB**	**Details**	**Resolution (Å)**	**Publication**
TolC	*E. coli*	1EK9	Trimer, closed state. C-terminal 43 amino acids removed.	2.1	Koronakis et al., 2000
MacA	*A. actino*	4DK0	Functions as a hexamer, TMH removed.	3.5	Xu et al., 2012
	*E. coli*	3FPP	Functions as a hexamer, TMH removed. MP present but not resolved.	2.99	Yum et al., 2009
	*S. pneu*	5XU0	SeMet, functions as a hexamer, MP domain and TMH removed.	2.95	Yang et al., 2018
MacB	*A. actino*	5LIL	ATPɤS-bound dimer (P2_1_).	3.35	Crow et al., 2017
		5LJ6	ATP-bound dimer (P6_5_22).	3.9	Crow et al., 2017
		5LJ7	ATP-bound dimer (P2_1_).	3.25	Crow et al., 2017
	*A. baumannii*	5GKO	SeMet labeled, nucleotide-free dimer.	3.39	Okada et al., 2017
		5WS4	ADPβS-bound dimer.	3.4	Okada et al., 2017
	*S. pneu*	5XU1	Nucleotide-free dimer.	3.3	Yang et al., 2018
MacAB-TolC	*E. coli*	5NIL	Nucleotide-free MacA_6_MacB_2_TolC_3_ assembly Cryo-EM (MacB region).	5.3	Fitzpatrick et al., 2017
	*E. coli*	5NIK	Nucleotide-free MacA_6_MacB_2_TolC_3_ assembly. Cryo-EM (MacA-TolC region). TolC in the open state.	3.3	Fitzpatrick et al., 2017
MacB periplasmic domains	*A. actino*	3FTJ	Monomer, functions as dimer in full-length protein.	2.0	Xu et al., 2009
	*E. coli*	5C59	SeMet monomer (P2_1_ β = 99.7°). Functions as a dimer in full-length protein.	3.0	Ha and Kim, unpublished
	*E. coli*	5LJ8	Monomer, extended conformation (P2_1_ β = 92.9°). Functions as a dimer in full-length protein.	1.95	Crow et al., 2017
MacB cytoplasmic NBD	*E. coli*	5LJ9	Nucleotide-free monomer. (C222_1_), functions as a dimer in full-length protein.	2.3	Crow et al., 2017
	*E. coli*	5LJA	Nucleotide-free monomer (P6_1_22), functions as a dimer in full-length protein.	2.4	Crow et al., 2017

*A. actino, Aggregatibacter actinomycetemcomitans; S. pneu, Streptococcus pneumoniae; SeMet, selenomethionine labeled; MP, Membrane proximal; TMH, Transmembrane helix*.

### The outer membrane exit duct, TolC

The first structural component of a TEP solved was the TolC outer membrane channel (Koronakis et al., 2000; Figure 3). Three TolC monomers trimerise to form a 12-stranded β-barrel and a 100 Å long α-helical tunnel extending down into the periplasm. An equatorial domain comprising the N- and C-termini forms a belt around the middle of the α-helical domain. This α-tunnel is composed of six pairs of coiled coils packing together, and has an approximate diameter of 35 Å for almost its entire length. At the bottom of the channel, three aspartate residues, one from each monomer, form a constriction of ~4Å effectively closing the channel at the periplasmic side of the membrane. This closed conformation of the tunnel is constrained by an inter- and intra-monomer network of hydrogen bonds and salt bridges. Crystal structures of TolC variants, in which this stabilizing network was disrupted by one or more point mutations, suggested how the duct opens to allow passage of substrate (Bavro et al., 2008; Pei et al., 2011). Subsequently, pseudoatomic cryo-EM structures of the AcrAB-TolC pump, in the presence and absence of substrate, revealed the iris-like opening of the periplasmic constriction of wild-type TolC (Wang et al., 2017). TolC homologs from other organisms are divergent in sequence and exhibit variation in the structure of the equatorial domain (Akama et al., 2004; Federici et al., 2005; Kulathila et al., 2011; Su et al., 2014; Guan et al., 2015; Yonehara et al., 2016). However, the same β-barrel and α-tunnel structure is evident suggesting the gross topology is likely to be conserved in all outer membrane efflux channels from Gram-negative bacteria.

### The periplasmic adaptor protein, MacA

Structures of *E. coli* and *Aggregatibacter actinomycetemcomitans* MacA have both been solved by X-ray crystallography (Yum et al., 2009; Xu et al., 2012). Like the RND pump adaptors, AcrA (Mikolosko et al., 2006) and MexA (Higgins et al., 2004), the MacA adaptor comprises helical hairpin, lipoyl, β-barrel and MP domains. The native MacA protein also has an N-terminal TMH, which distinguishes it from other adaptors, including AcrA and MexA, typically anchored to the inner membrane by an N-terminal lipoyl group (Hinchliffe et al., 2013). However, the MacA crystal structures were determined using protein constructs lacking this N-terminal TMH. Both *E. coli* and *A. actinomycetemcomitans* MacA crystallized as hexamers without additional stabilization using chemical cross-linking reagents or engineered disulfide bonds (Yum et al., 2009; Xu et al., 2012). The MacA hexamer resembles an inverted funnel with the 70 Å stem formed by the hairpin domains and a wider mouth formed by association of the lipoyl and β-barrel domains. The cryo-EM structure of *E. coli* MacA within the context of the assembled pump revealed a similar hexameric organization (Fitzpatrick et al., 2017). Notably, six lipoyl domain loops, one from each monomer, project into the center of the MacA channel. The resulting constriction is stabilized by inter-protomer hydrogen bonds between glutamine residues at the tip of the loop (Fitzpatrick et al., 2017). These glutamine residues are conserved in MacA proteins (Yum et al., 2009; Fitzpatrick et al., 2017) but replacement with alanine did not affect MacAB conferred erythromycin resistance (Fitzpatrick et al., 2017). Instead, steered molecular dynamics simulations suggested that the lipoyl domain loops favor unidirectional movement of erythromycin through the MacA channel. The construction in the MacA hexamer could therefore act as a gate to regulate flow of substrates through the assembled efflux pump (Fitzpatrick et al., 2017).

### The inner membrane ABC transporter, MacB

The structure of the isolated periplasmic domain of the monomeric MacB periplasmic domain was solved almost 10 years ago revealing the presence of two subdomains (Xu et al., 2009). However, without the context of the membrane, it was difficult to infer details of the transport mechanism. Now, a series of structures of full-length MacB have helped reveal molecular details of its operation. MacB from *A. actinomycetemcomitans* and *A. baumannii* were crystallized in ATP- and ADP-bound forms, respectively (Crow et al., 2017; Okada et al., 2017; Figure 3). MacB crystallized as a dimer consistent with analytical ultracentrifugation and atomic force microscopy studies (Lin et al., 2009). Each MacB monomer comprises an N-terminal NBD, four TMHs and a large periplasmic domain between TMH1 and TMH2. Helical extensions of TMH1 and TMH2 from each monomer form a four helix bundle that elevates the periplasmic domain above the plane of the membrane, giving a mushroom-like appearance. TMH3 and TMH4 pack on the outside of the helical bundle and are linked by a short extracytoplasmic loop which has been referred to as the “shoulder” (Crow et al., 2017). The cytoplasmic N-terminus of TM1 is connected to the NBDs by an amphipathic helix running parallel to the membrane surface and a “skirting loop.” The major coupling helix is located on the cytoplasmic side of the membrane between TM2 and TM3, and provides a means of communicating conformational changes from the NBDs to the TMDs, as suggested for other ABC transporters. A second helix at the C-terminus, the minor coupling helix, also makes contact with the NBD. Deletion of the minor coupling helix had a modest impact on activity of *E. coli* MacB (Crow et al., 2017) but a greater effect on *A. baumannii* MacB mediated macrolide resistance (Okada et al., 2017), which may reflect differences in the assays used.

**Figure 4:**
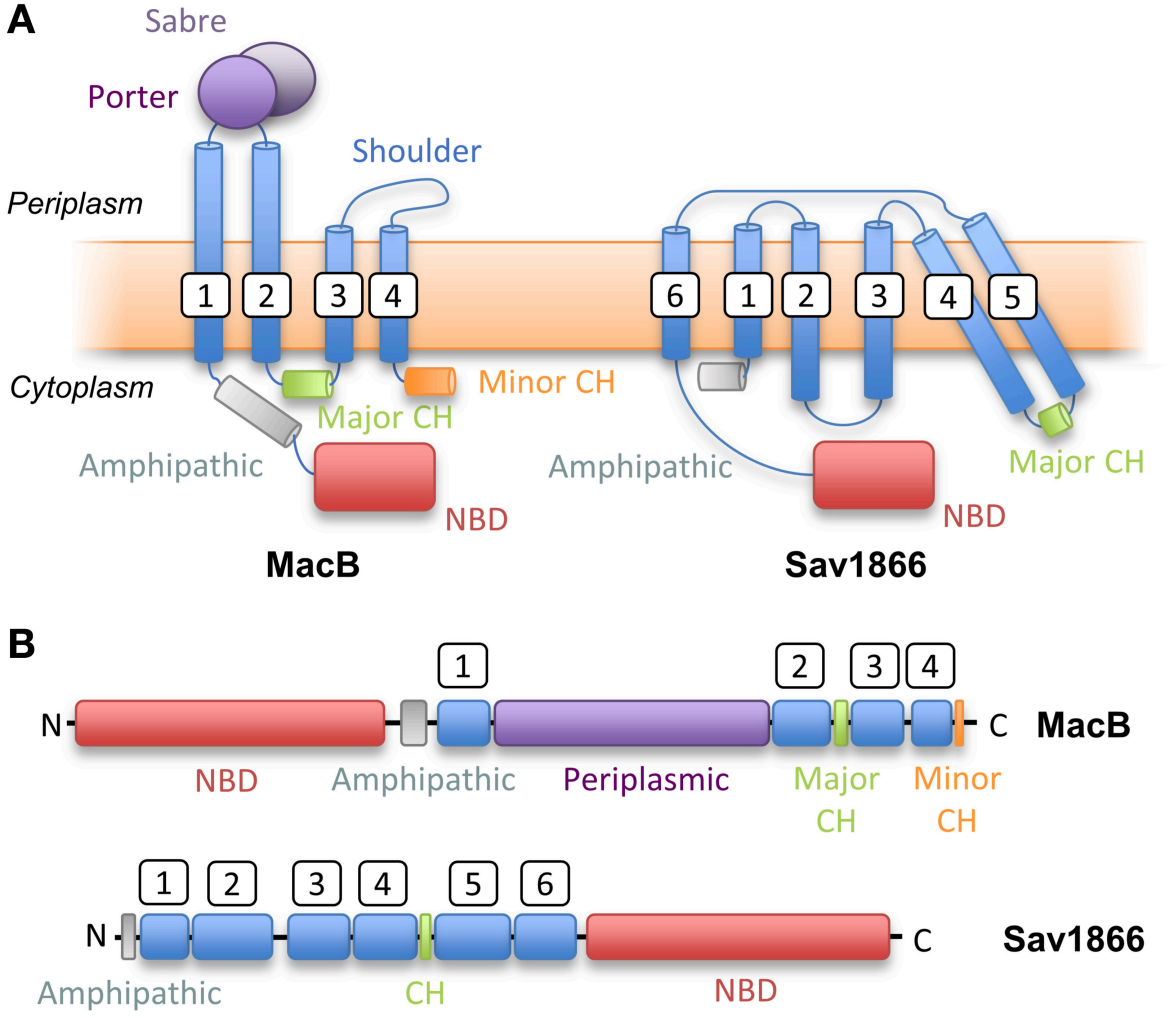
Topological comparison of MacB (a Type VII ABC transporter) with classical exporter Sav1866 (a Type IV ABC transporter). **(A)** Topology diagrams based on structures of MacB and Sav1866. **(B)** Linear domain arrangements for MacB (upper) and Sav1866 (lower). CH, Coupling Helix; NBD, Nucleotide-Binding Domain. White boxes indicate transmembrane helix numbering.

The two subdomains of the MacB periplasmic domain are the Porter, named for structural similarity to a domain of AcrB, and the Sabre named for the acronym: Small, alpha/beta rich, extracytoplasmic. The Sabre domain is formed from a contiguous stretch of residues (347–465 in *A. actinomycetemcomitans* MacB) while the Porter domain is formed from two β-α-β motifs found on either side of the Sabre domain (residues 306–346 and 466–503). The Porter domain is directly connected to both stalk helices of the MacB monomer. The same fold is evident in the ADP-bound *A. baumannii* MacB structure although there are striking differences in conformation (see mechanotransmission section below). The crystal structures reveal that the architecture of MacB is clearly distinct from classic ABC exporters such Sav1866 (Figure 4). Like other multidrug exporters, Sav1866 is characterized by a 6-TMH topology, C-terminally fused NBD, and a coupling helix located between TMH4 and TMH5 of each monomer that reciprocally “cross-over” to engage the NBDs. MacB, in contrast, has four TMHs, an N-terminally fused NBD, a large periplasmic domain and a major coupling helix that engages the NBDs in intra-molecular fashion.

### The assembled MacAB-TolC tripartite pump

The significant technical challenge of isolating an assembled MacAB-TolC pump and maintaining it through the purification procedure was achieved by creating a fusion of MacA to the C-terminus of MacB, and subsequently by introducing specific cysteine residues into MacA and MacB to stabilize their interaction with disulfide bonds (Fitzpatrick et al., 2017). Crystal structures of *E. coli* MacA (Yum et al., 2009), *E. coli* TolC (Koronakis et al., 2000) were already available. Docking these, and a homology model of *E. coli* MacB based on the *A. baumannii* MacB structure (Okada et al., 2017), into a cryo-EM map enabled the structure of the *E. coli* MacAB-TolC assembly to be solved (Fitzpatrick et al., 2017) (Figure 3). The assembled tripartite pump comprises a single MacB dimer, MacA hexamer and the trimeric TolC exit duct. The complete MacAB-TolC assembly is approximately 320 Å long, comparable to the size of the AcrAB-TolC pump (Du et al., 2014; Daury et al., 2016).

The structure reveals how the three components interact in the context of an assembled pump (Fitzpatrick et al., 2017). The tips of the MacA hairpin domains intermesh with the periplasmic ends of the TolC coiled coils. These “tip to tip” interactions were first suggested on the basis of *in vitro* binding experiments (Xu et al., 2010), and EM analysis of a complex of *E. coli* MacA and a hybrid protein bearing the tip regions of the TolC helical barrel (Xu et al., 2011). Association with MacA stabilizes the TolC trimer in an open state in which the aspartates at the periplasmic constriction are almost 30 Å apart. This results in a continuous channel from the top of TolC down to the lipoyl loop constriction in MacA. Adaptor interactions with the periplasmic domain of MacB are mediated by the MacA β-barrel and MP domains. The annulus of MacA β-barrel domains sit on top of MacB with three MacA monomers contacting each MacB. The ring of MacA MP domains makes extensive contacts with the MacB periplasmic domain consistent with previous biochemical studies (Modali and Zgurskaya, 2011). Two MacA MP domains contact the Sabre, and one interacts with the Porter subdomain of each MacB monomer. The MacA hairpin and lipoyl domains do not contact MacB (Fitzpatrick et al., 2017).

The MacB structure within the assembled complex resembles that of the ADP-bound *A. baumannii* structure. The NBDs are separated and the MacB stalk helices bend away from each other leading to a separation of the periplasmic domains and formation of a periplasmically accessible cavity between MacB and MacA (Fitzpatrick et al., 2017).

## The mechanotransmission mechanism of MacB

**Figure 5:**
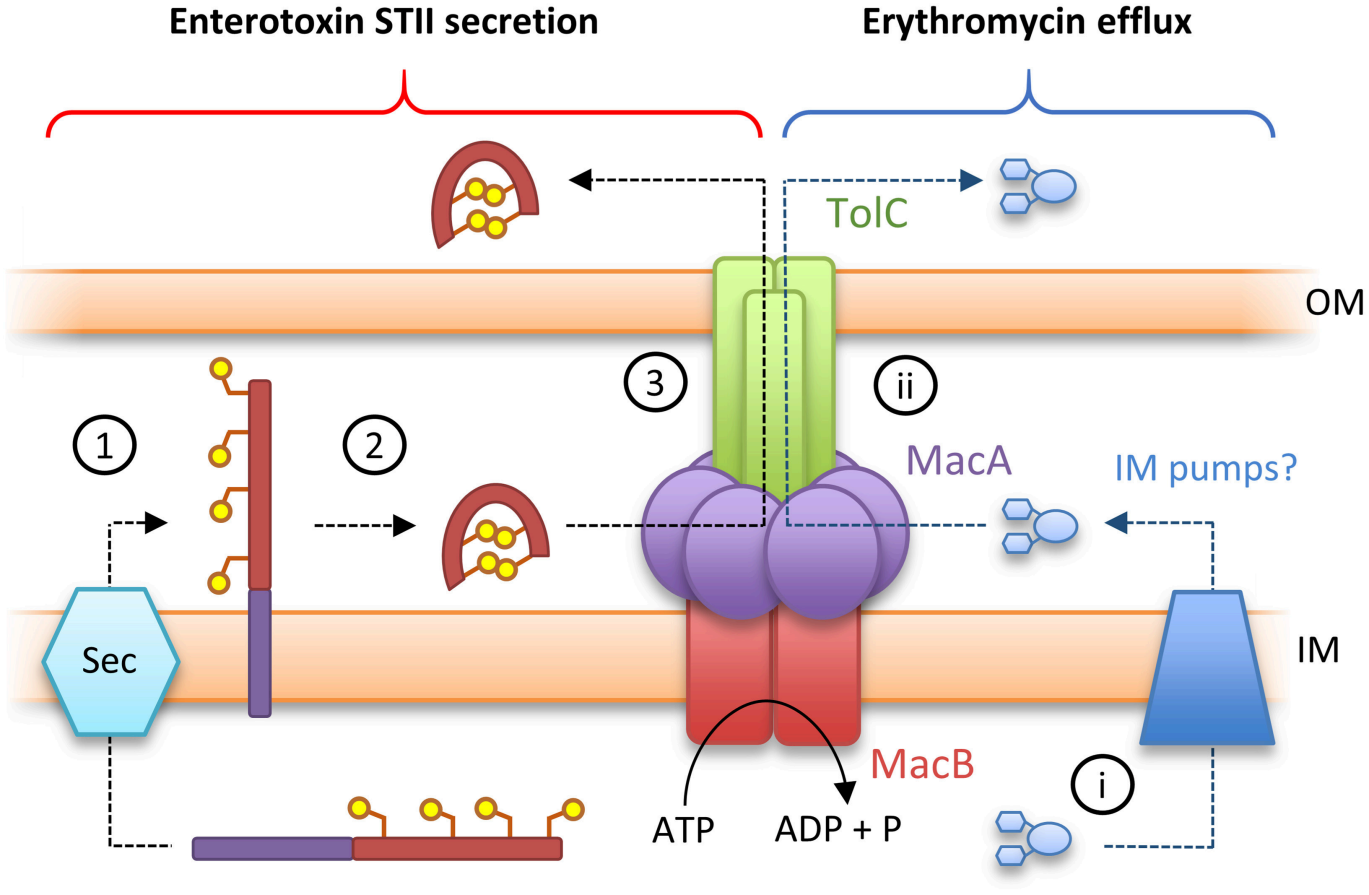
Toxin secretion and antibiotic resistance by the MacAB-TolC tripartite efflux pump. **(Left)** A model for enterotoxin STII secretion: (1) Enterotoxin STII is produced ribosomally in the cytoplasm and transported to the periplasm by the Sec machinery. (2) The N-terminal secretion signal (*purple bar*) is cleaved and two disulfide bonds are incorporated by DsbA. (3) Export of enterotoxin across the bacterial outer membrane is driven by the MacAB-TolC pump. **(Right)** A model for antibiotic detoxification: (i) antibiotics (here erythromycin) in the cytoplasm are directed to periplasm by inner membrane pumps. (ii) Antibiotics in the periplasm are driven across the bacterial outer membrane by MacAB-TolC. Adapted from Crow et al. (2017).

Canonical ABC transporters provide a transmembrane substrate pathway in the form of a vestibule or cavity located between the two halves of the dimer interface (Choudhury et al., 2014). Analysis of the MacB structures described above indicate there is not a sufficient channel to provide a transmembrane pathway for substrate in either the ATP or nucleotide-free state (Crow et al., 2017). Furthermore, a functional MacAB-TolC pump is required to export peptide substrate enterotoxin STII (Yamanaka et al., 2008; Crow et al., 2017). STII is exported to the periplasm by the Sec system, and requires the action of the periplasmically located Dsb system to catalyze the formation of its two disulfide bonds (Foreman et al., 1995). Taken together, these data suggest MacB does not transport substrates across the inner membrane but instead accepts substrates in the periplasm and, in concert with MacA, ejects them across the outer membrane via the TolC exit duct (Figure 5). This mechanism is functionally akin to the “periplasmic vacuum cleaner” model, proposed for RND-type transport pumps, in which substrates are bound in the periplasm and ejected across the outer membrane (Aires and Nikaido, 2005). The network is completed by standalone transporters in the inner membrane which remove substrates from the cytoplasm (Tal and Schuldiner, 2009).

**Figure 6:**
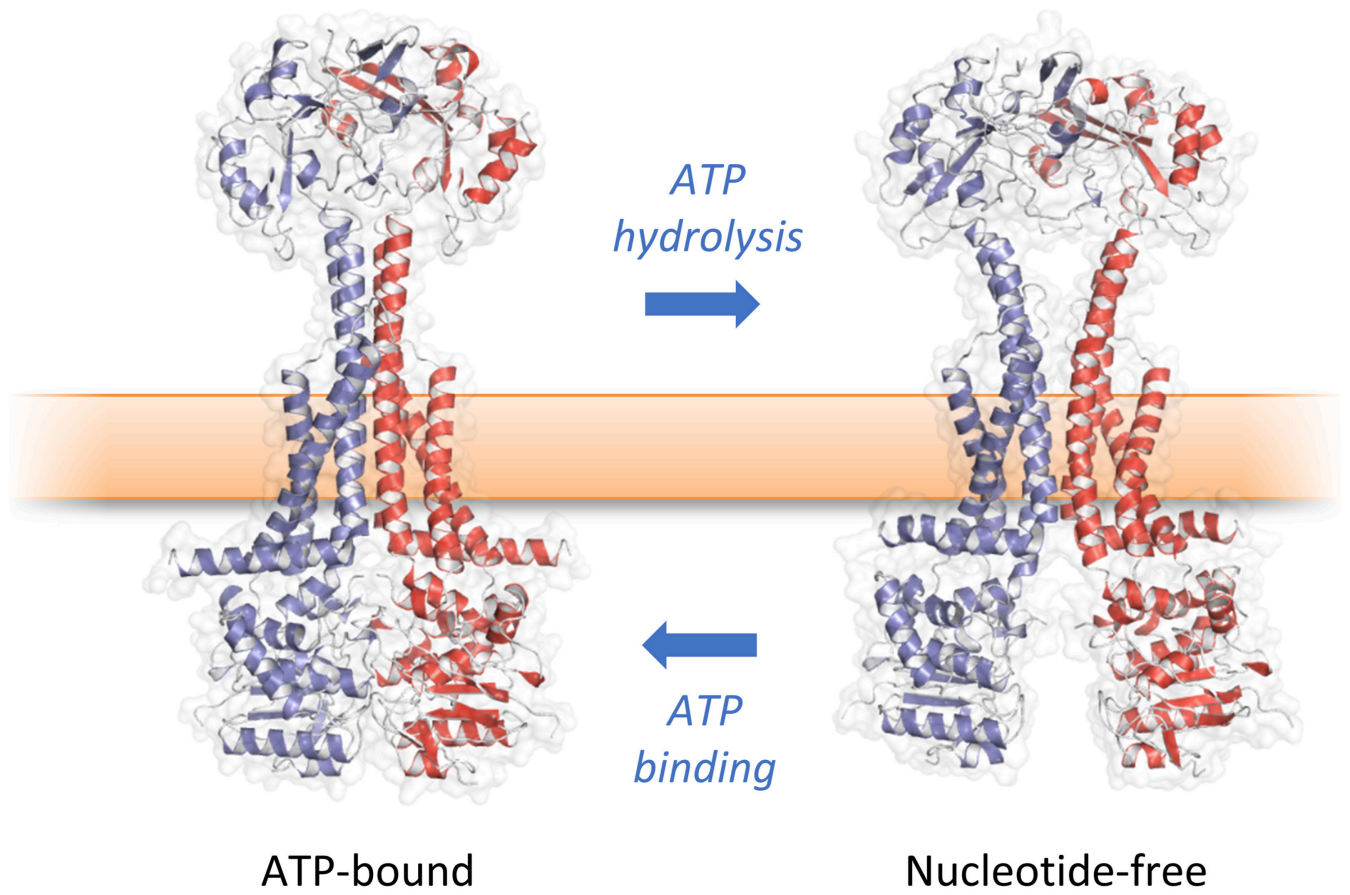
Mechanotransmission mechanism of MacB. ATP binding and hydrolysis cause large, transmembrane conformational changes in MacB structure. Rather than transporting substrates across the inner membrane, MacB-like proteins coordinate reversible dimerization of their NBDs with periplasmic conformational changes. TEP-forming MacB homologs use periplasmic conformational change to drive substrates across the bacterial outer membrane via TolC-like exit ducts. MacB homologs that do not form TEPs are proposed to use similar motions during lipoprotein trafficking and transmembrane signaling. Adapted from Crow et al. (2017).

How then do ATP-induced changes in the cytoplasmic NBDs affect the periplasmic domains of MacB? Comparison of the ATP-bound and free forms of MacB reveal long-range conformational changes in the transmembrane helices, stalk and periplasmic domains (Crow et al., 2017; Fitzpatrick et al., 2017; Okada et al., 2017). In the nucleotide-free form, ATP binding induces dimerization of the NBDs causing the major coupling helix to push upwards on TM2. The resultant “zipping up” of the transmembrane helices into a rigid 4-helix bundle brings the periplasmic domains of each monomer together, eliminating the cavity between them (Figure 6). We term these long-range movements mechanotransmission, as ATP hydrolysis is not used to transport a substrate across the membrane in which the ABC protein resides, but instead to transmit conformational changes from cytoplasmic NBDs to achieve useful work in the periplasm. The importance of the relative movements of the stalk helices was underlined by a severe reduction in MacB function *in vivo* when the helices were locked together with a disulfide bond (Crow et al., 2017).

**Figure 7:**
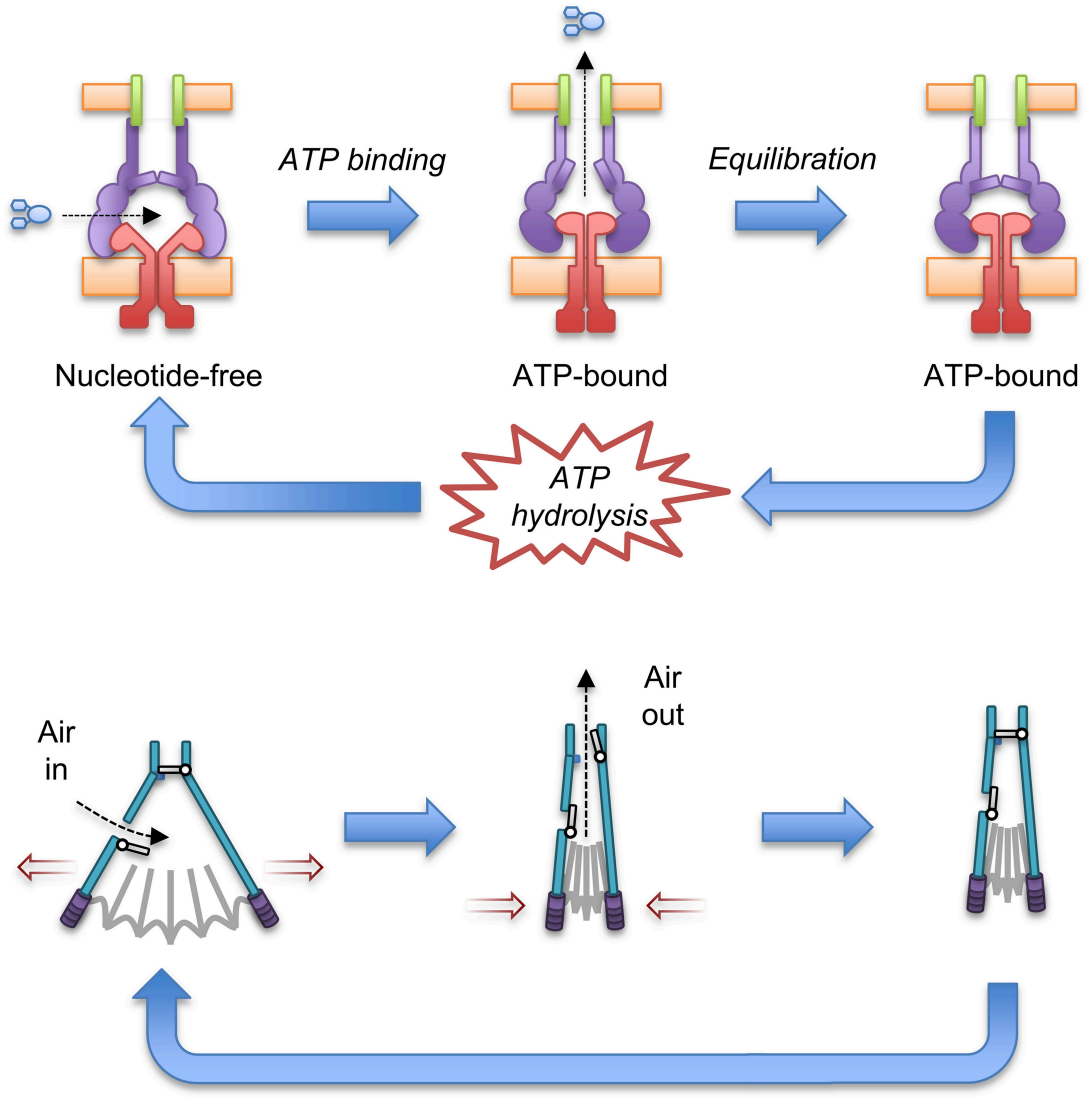
A molecular bellows mechanism for the MacAB-TolC pump driven by mechanotransmission. The proposed catalytic cycle of the MacAB-TolC efflux pump **(Top)** is shown in comparison with the operation of a fireplace bellows **(Bottom)**. Substrates are driven out by mechanotransmission-induced changes in the MacB periplasmic domains and prevented from flowing back into the periplasm by a valve in MacA. The individual pump components are colored: MacB (red), MacA (purple), TolC (green) and substrate (blue). Adapted from Crow et al. (2017).

Docking of the ATP-bound MacB form into the cryo-EM MacAB-TolC structure has led to the suggestion that MacB could harness mechanotransmission to operate the assembled pump as a molecular bellows (Crow et al., 2017; Figure 7). The MacA hexamer accommodates the MacB periplasmic domain in both open (ATP-free) and closed (ATP-bound) conformations. Initially, the pump is in a nucleotide-free state in which the stalk helices and periplasmic domains of MacB are separated. Consequently, substrates can enter the cavity at the interface of MacB and MacA. ATP binding brings the NBDs together and the mechanotransmission mechanism results in tight association of the stalk helices and MacB periplasmic domains. The ensuing reduction in the cavity volume creates pressure which forces the substrates upwards into the MacA channel and through the MacA gate described above. Once the pressure is equalized, the gate closes, preventing backflow of substrates. ATP hydrolysis then resets the pump, with the MacB stalk helices moving apart allowing substrates to bind and another round of efflux to proceed. The proposed model requires a mechanism to prevent substrate egress back through MacB. Cryo-EM analysis of the assembled AcrAB-TolC TEP revealed a ~10 Å contraction along the long axis of the pump in the substrate-bound state (Wang et al., 2017). A similar contraction of the MacAB-TolC TEP could ensure that MacA packs more tightly around MacB to ensure substrates are driven out through MacA/TolC rather than escaping into the periplasm. Clearly, structures of a substrate-bound state of the MacB-driven TEP will be invaluable in determining the precise details of its mechanism.

Little is known about how substrates are recognized by MacB. Unexplained electron density at the cleft between the MacB periplamic domains in the assembled pump could represent an endogenous substrate (Fitzpatrick et al., 2017), but details remain obscure. Mutagenesis of a patch of predominantly hydrophobic residues, on the interior surface of the periplasmic domain, reduced MacB conferred resistance to erythromycin, bacitracin and colistin. This patch constitutes a plausible substrate binding domain (Crow et al., 2017). However, it is still unclear whether antibiotics are directly effluxed or resistance is mediated as an indirect effect of transport of another substrate. Zgurkskaya and colleagues demonstrated *E. coli* MacA co-purified with lipopolysaccharide and suggested the native substrate could be a glycolipid (Lu and Zgurskaya, 2013).

Biochemical data suggests that MacA is able to influence the ATPase activity of MacB and may have a role in communicating the presence or absence of substrate in the periplasm to the cytoplasmic NBDs (Tikhonova et al., 2007; Modali and Zgurskaya, 2011; Lu and Zgurskaya, 2012). Deletion of the cytoplasmic region, or N-terminal MacA TMH, compromised the MacA-mediated stimulation of MacB ATPase activity *in vitro* and prevented MacAB mediated macrolide resistance *in vivo* (Tikhonova et al., 2007). The structural basis for ATPase stimulation remains unclear, because the MacA TMH was not resolved in the MacAB-TolC cryo-EM structure (Fitzpatrick et al., 2017).

## MacB family mediated antibiotic resistance in gram-positive bacteria

The classic view of adaptor proteins is that they serve to bridge the inner and outer membrane components of TEPs. Bioinformatic analysis suggesting the widespread presence of adaptor proteins in Gram-positive bacteria was therefore somewhat unanticipated (Harley et al., 2000; Zgurskaya et al., 2009). Many of the genes encoding these Gram-positive adaptor proteins are found in operons with homologs of *macB*. The first of these systems to be characterized, YknXYZ, is responsible for protection from the endogenous toxin, sporulation delaying peptide (SDP). The release of SDP causes lysis of nearby cells to provide nutrients for producing cells to complete sporulation – a process that has been described as bacterial cannibalism. Mature SDP is a 42 amino acid peptide with one disulfide bond (Liu et al., 2010). Structures of the adaptor protein YknX (Xu et al., 2017) reveal that it has the same fold and hexameric arrangement as *E. coli* MacA (Yum et al., 2009; Fitzpatrick et al., 2017). Similarly, the permease component YknZ is clearly homologous to the transmembrane and periplasmic domain of MacB (Xu et al., 2016; Fitzpatrick et al., 2017), while YknY encodes a cytoplasmic NBD. Sec-dependence of SDP export suggests that like MacB, YknXYZ probably mediates its protective effect from the extracytoplasmic side of the membrane—perhaps by keeping SDP away from the bilayer. Interestingly, a co-transcribed membrane protein, YknW, may influence the oligomerisation and/or conformational state of the adaptor YknX, but its role remains enigmatic (Yamada et al., 2012).

In *Enterococcus faecalis*, a MacB-type transporter and cognate adaptor are required for protection against endogenously produced AS-48, a 70 amino acid cyclic peptide antibiotic (González et al., 2000). A 10 gene cluster organized into two operons is associated with AS-48 biosynthesis. The first operon encodes genes responsible for AS-48 production and export across the cytoplasmic membrane while the second operon, AS-48EFGH, is important for resistance to exogenous AS-48 (Diaz et al., 2003). Together, AS-48G (NBD) and AS-48H (TMD) encode a MacB-like ABC transporter with an extracytoplasmic domain of approximately 230 amino acids. AS-48E is predicted to contain four TMHs and cytoplasmic N- and C-termini. Further investigation is required to establish the role of AS-48E and whether it influences the AS48-FGH pump activity in a similar manner to YknW in the SDP-exporting pump described above.

A global analysis of *Streptococcus pneumoniae* D39 gene expression showed upregulation of an operon (Sp0785-0787) in response to bacitracin and the human defensin LL-37. Deletion of this operon increased susceptibility to LL-37 and lincomycin (Majchrzykiewicz et al., 2010). Analysis of these genes suggest they encode a periplasmic adaptor protein and transporter homologous to MacB. Very recently, structures of the equivalent proteins from *S. pneumoniae* R6 (Spr0693-0695) were obtained (Yang et al., 2018). These structures reveal remarkable structural similarity to the Gram-negative proteins already crystallized. *S. pneumoniae* MacA (hereafter SpMacA) was crystallized in the absence of the MP domain and is made up of characteristic β-barrel, lipoyl and α-helical hairpin domains. A lipoyl domain loop from each monomer projects into the SpMacA channel in a similar fashion observed in the *E. coli* MacA structure (Fitzpatrick et al., 2017). However, the residue at the tip is not conserved and the physiological role of the SpMacA loop was not tested (Yang et al., 2018). SpMacB is remarkably similar to the Gram-negative MacB structures (Crow et al., 2017; Fitzpatrick et al., 2017; Okada et al., 2017; Yang et al., 2018). It was crystallized in the presence of the non-hydrolysable ATP analog AMP-PNP although this was not resolved in the structure, and the arrangement of the stalk helices is more similar to the ADP-bound or nucleotide-free form (Fitzpatrick et al., 2017; Okada et al., 2017; Yang et al., 2018). As with *E. coli* MacAB (Tikhonova et al., 2007), the ATPase activity of SpMacB reconstituted in proteoliposomes was stimulated by co-reconstitution with full-length SpMacA, but not by variants lacking the cytoplasmic region and N-terminal TMH (Yang et al., 2018).

In these Gram-positive systems, an adaptor protein is present but in the absence of an outer membrane, what is its role? Cryo-EM studies of vitrified cell samples reveal a discrete zone between the inner membrane and peptidoglycan akin to a Gram-positive “periplasm” (Matias and Beveridge, 2006; Zuber et al., 2006). The presence of these adaptors could therefore form channels to remove xenobiotics from this space, and prevent their immediate re-association with the membrane. The height of the SpMacA channel is consistent with the dimensions of this periplasm-like zone (Yang et al., 2018) and purified YknX adaptor is able to bind peptidoglycan (Xu et al., 2017). Further analysis of Gram-positive MacAB-like assemblies is vital to understand their roles *in vivo*.

## Sensing and resisting the threat of lantibiotics using the MacB architecture

Lantibiotics are post-translationally modified peptides containing the polycyclic thioether amino acids lanthionine or methyllanthionine e.g., bacitracin and nisin (Draper et al., 2015). They are produced by low G+C Gram-positive bacteria and typically interfere with cell wall/peptidoglycan biosynthesis by binding to precursors such as undecaprenyl-pyrophosphate (UPP) and Lipid II (Draper et al., 2015). Multiple methods of resistance to lantibiotics exist, but intriguingly one class of resistance proteins are ABC transporters homologous to MacB proteins. These systems are exemplified by BceAB of *B. subtilis* but multiple homologs in other Gram-positive bacteria have been characterized including *Streptococcus mutans* MbrAB (Tsuda et al., 2002), *Listeria monocytogenes* AnrAB (Collins et al., 2010), *S. aureus* VraFG (Meehl et al., 2007), and *Streptococcus agalactiae* NsrFP (Khosa et al., 2013).

BceA encodes an ATPase domain, while BceB is a membrane permease with ten TMHs and a large extracellular domain between TMH7 and TMH8. The final four TMHs and intervening periplasmic domain are topologically similar to MacB. Intriguingly, the BceAB-type transporters appear to have coevolved with a two-component system (TCS) that works with BceAB to sense and respond to extracellular antibiotics (Coumes-Florens et al., 2011; Dintner et al., 2011). Typically, TCSs consist of a membrane intrinsic histidine kinase that phosphorylates a response regulator to control gene expression in response to an external stimulus. Two-hybrid analysis of the proteins suggest that BceB and BceS directly interact (Kallenberg et al., 2013; Dintner et al., 2014). However, unlike in classical TCS systems, the histidine kinase (BceS) cannot directly sense antibiotics and requires the presence of BceB and an active BceA ATPase domain to respond to bacitracin (Rietkötter et al., 2008). Two different mechanisms for BceB mediated detoxification have been suggested. Kingston and colleagues proposed that BceAB induces resistance by flipping UPP into the inner leaflet of the cytoplasmic membrane, thereby protecting it from bacitracin (Kingston et al., 2014). Conversely, Dintner and coworkers demonstrated purified BceAB complex could directly bind bacitracin-Zn^2+^, the active form of the peptide, with nanomolar affinity. They therefore suggested that BceAB mediates resistance by direct efflux of bacitracin itself (Dintner et al., 2014). Though the nature of the substrate *in vivo* remains unclear, BceAB is active in both the sensing and detoxification of bacitracin.

Analysis of the BceB sequence suggests a possible fusion of two MacB architectures in which the first periplasmic domain has been lost (Dintner et al., 2014). Consistent with this idea, bacterial two-hybrid analysis suggests BceB monomers do not interact. Furthermore, size-exclusion chromatography analysis of detergent-purified BceA:BceB complex suggested a 2:1 stoichiometry, although the purified complex did not have ATPase activity (Dintner et al., 2014). Mutations affecting both sensing, and detoxification, of bacitracin map to the C-terminal half of BceB, including TMH8 (Kallenberg et al., 2013). This helix is equivalent to TMH2 in MacB, suggesting a mechanotransmission-like mechanism could also underpin BceB action.

Unlike YknYZ and SpMacB discussed in the previous section, BceAB is not associated with an adaptor protein. However, interestingly, the activity of a BceAB homolog from *S. aureus* (VraDE) is modulated by a two-TMH membrane protein (VraH) that has cytoplasmic N- and C-termini. Two-hybrid analysis demonstrated VraH interacts with VraE and increases resistance to daptomycin and gallidermin but the mechanism of this modulation is not clear (Popella et al., 2016).

## Insights into the wider MacB family of ABC transporters

### TEP-forming MacB homologs

The structures and mechanisms detailed here are also likely to apply to MacB homologs operating as part of tripartite efflux pumps to export substrates other than antibiotics (Table 2). One such example is export of the siderophore pyoverdine by *Pseudomonas aeruginosa* PvdRT-OpmQ. Pyoverdine is matured by periplasmic enzymes, and mutants lacking PvdRT accumulate pyoverdine in the periplasm. These observations suggest that the MacB homolog PvdT mediates transport from the periplasm, not the cytoplasm (Hannauer et al., 2010). In other *Pseudomonas* species, homologs of MacAB are required for phytotoxin secretion (Cho and Kang, 2012). In these systems, the toxin biosynthetic operons include homologs of MacAB. By analogy with MacB, another pump would be required to export the non-ribosomally synthesized peptide across the cytoplasmic membrane into the periplasm, a role which could be fulfilled by SyrD-type ABC transporters (Quigley et al., 1993).

**Table 2:** Representative MacB superfamily TEPs.

**Protein**	**Organism(s)**	**Complex**	**Substrate(s)**	**Function(s)**	**References**
MacB	Gram-negative bacteria	MacAB-TolC (TEP)	Enterotoxin STII, Erythromycin, Colistin, Bacitracin, Kanamycin, Glycolipids, Protoporphyrin	Toxin secretion Antibiotic resistance Cell envelope biogenesis? Detoxification?	Kobayashi et al., 2001; Yamanaka et al., 2008; Lu and Zgurskaya, 2013; Turlin et al., 2014; Crow et al., 2017
PvdT	*Pseudomonas aeruginosa*	PvdRT-OpmQ (TEP)	Pyoverdine	Siderophore export Virulence?	Imperi et al., 2009; Hannauer et al., 2012
AatP	Enteroaggregative *E. coli* (EAEC)	AatABCP (TEP)	Dispersin	Biofilm dispersal signaling	Nishi et al., 2003; Velarde et al., 2007
DevC	*Anabaena* sp.	DevABC-TolC	Glycolipids	Heterocyst envelope biogenesis	Staron et al., 2011, 2014
CexP	Enterotoxigenic *E. coli* (ETEC)	CexPABC	CexE?	CexE secretion?	Pilonieta et al., 2007

The MacB-type ABC transporter DevCA, adaptor protein DevB and TolC homolog HgD form a TEP responsible for glycolipid export underpinning heterocyst formation in the nitrogen fixating cyanobacterium *Anabaena*. Deletion of the N-terminus of DevB prevents substrate export, but not association with DevCA, suggesting the cytoplasmic region of the adaptor protein can control the activity of the pump as proposed for *E. coli* MacAB-TolC (Staron et al., 2011, 2014).

Enteroaggregative *E. coli* express a virulence plasmid-encoded MacB based TEP dedicated to the export of dispersin, a positively-charged, surface-associated protein, that prevents bacterial aggregation (Nishi et al., 2003). A similar TEP exports the dispersin homolog, CexE, from enterotoxigenic *E. coli* (Pilonieta et al., 2007). The structure of 10 kDa dispersin is made up of two antiparallel 3-stranded beta-sheets with decorating α-helices at either end, resulting in a 20 Å diameter, 50 Å long “bullet” shape (Velarde et al., 2007). The narrowest dimension is consistent with transport through the periplasmic cavity of MacB (and the lumen of the TolC exit duct) but may represent the upper size-bound for folded substrates that are transported by MacB-dependent TEPs.

### TEP-independent MacB homologs

The MacB architecture is also found in proteins that operate independently of adaptor proteins/TEPs (Table 3). For example, LolCDE, an ABC transporter with the same topological organization as MacB, underpins lipoprotein trafficking in Gram-negative bacteria. Lipoproteins destined for the outer membrane are first transported across the cytoplasmic membrane by the Sec system, and then successively acylated by membrane intrinsic machineries (Narita and Tokuda, 2017). LolCDE then extracts the lipid moiety of lipoproteins from the outer leaflet of the inner membrane and passes it to the periplasmic chaperone LolA for delivery to the outer membrane. LolD encodes an NBD, while LolC and LolE both have the 4-TM helix MacB type architecture (Yakushi et al., 2000; Narita and Tokuda, 2017). The structure of the periplasmic domain of LolC revealed a fold homologous to MacB, suggesting that lipoprotein extraction could be performed by the LolCDE complex using a mechanotransmission mechanism (Crow et al., 2017).

**Table 3:** Representative non-TEP forming homologs of the MacB ABC transporter.

**Protein**	**Organism(s)**	**Complex**	**Substrate(s)**	**Function(s)**	**References**
LolC, LolE, LolF	Gram-negative bacteria	LolCDE or LolDF	Lipoproteins	Lipoprotein extraction and trafficking	Yakushi et al., 2000; Narita and Tokuda, 2017
FtsX	Gram-positive and Gram-negative bacteria	FtsEX	–	Cell division	Yang et al., 2011; Mavrici et al., 2014; Du et al., 2016
YknZ	*Bacillus amyloliquefaciens*	YknXYZ	Antimicrobial peptide?	Detoxification?	Yamada et al., 2012; Xu et al., 2017
BceB	*Bacillus subtilis*	BceABS	Bacitracin,	Sensing & detoxification	Dintner et al., 2014
HrtB	Gram-positives	HrtAB	Heme?	Detoxification?	Stauff et al., 2008; Bibb and Schmitt, 2010
AS-48H	*Enterococcus faecalis*	AS-48EFGH	Mature AS-48?	Bacteriocin AS-48 export?	Diaz et al., 2003

The MacB architecture is also found in FtsEX which is required for efficient cell division in Gram-negative bacteria (Schmidt et al., 2004; Yang et al., 2011; Du et al., 2016), sporulation in *Bacillus* (Garti-Levi et al., 2008) and survival of mycobacteria (Mavrici et al., 2014) and *Streptococcus* (Sham et al., 2011). In these organisms, the FtsEX complex is proposed to regulate the activity of extracytoplasmic cell wall amidases in the final stages of cell division. The periplasmic domain of *Mycobacterium tuberculosis* FtsX lacks a significant Sabre subdomain, but the Porter subdomain is remarkably similar to that of *E. coli* MacB (Mavrici et al., 2014; Crow et al., 2017). The absence of the Sabre and conservation of the Porter subdomain in FtsEX raises interesting questions regarding the role of these subdomains in MacB and other Type VII ABC transporters, including LolCDE and FtsEX. As far as we are aware, the Porter subdomain is present in all members of the Type VII ABC superfamily and is likely an intrinsic part of the mechanotransmission apparatus. The role of the Sabre subdomain is less obvious but it may be adapted to carry out specific tasks in different proteins.

HrtAB is another ABC transporter homologous to MacB found throughout Gram-positive bacteria. The HrtAB pair were initially proposed to protect cells from the toxic effect of high concentrations of heme by removing it from the cytoplasm (Stauff et al., 2008; Bibb and Schmitt, 2010). Direct transfer of substrates from the cytoplasm to the extracellular space has not yet been demonstrated, and more recent studies suggest HrtAB removes heme from the membrane in *S. aureus* (Wakeman et al., 2012) and *L. lactis* (Joubert et al., 2014). Consistent with this idea, mutation of two conserved tyrosine residues in the periplasmic domain abrogated HrtAB mediated tolerance of heme stress. Comparison of HrtB with MacB suggests these residues map to the top of the stalk, and so it is tempting to speculate that they could co-ordinate heme during transport. Further study of HrtAB is essential to assess whether this assembly receives substrates from the cytoplasm or not, and whether it can transport such substrates across the inner membrane.

**Figure 8:**
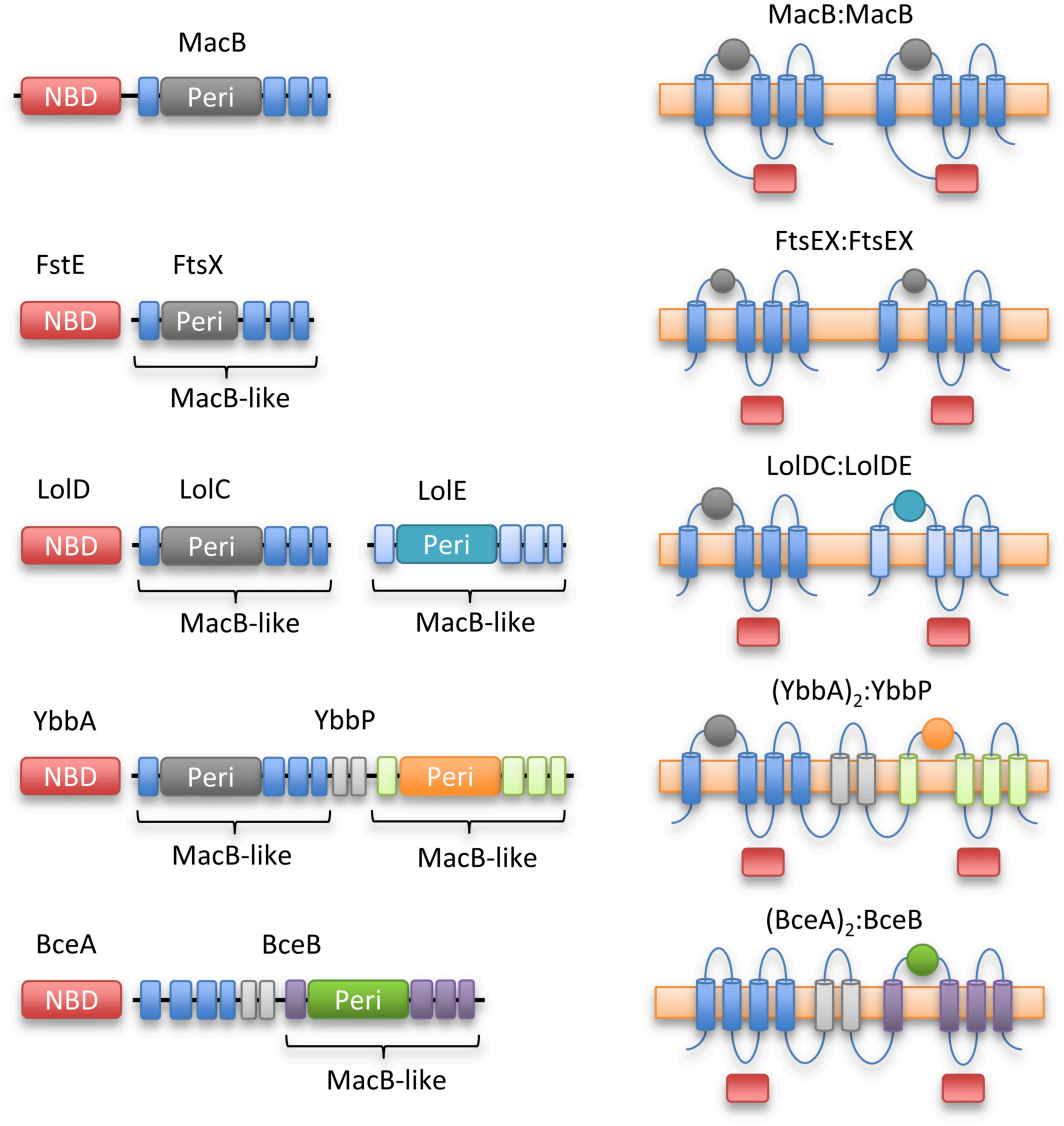
Topological organization of MacB family members. Linear domain arrangement **(Left)** and transmembrane topologies **(Right)** are shown for MacB and four representative homologs. From top to bottom, macrolide and toxin transporter MacB, cell division protein FtsEX, lipoprotein trafficking LolCDE, YbbAP (unknown function), bacitracin sensing and efflux protein BceAB. MacB type ABC transporters share a distinctive transmembrane topology although NBDs are not always fused to transmembrane encoding domain and some families exist as apparent fusions of two half-transporters. Sequence identity between the periplasmic domains of each Type VII motif is low, but the extracellular domain of *Mycobacterium tuberculosis* FtsEX (Mavrici et al., 2014) also contains a Porter subdomain suggesting this could be a common element of Type VII ABC proteins.

YbbP is an *E. coli* protein of unknown function that appears to represent a fusion of two MacB permease units with two linking TMHs. Surveying this, and other homologs, reveals that the MacB 4-TMH architecture may be organized in different ways. The NBDs may be fused to the TMDs or encoded in a separate polypeptide. Similarly, the transmembrane domains may organize as homodimer (MacB and FtsX) or heterodimer of individual units (LolCE). Alternatively, the two permease domains may be fused into a single polypeptide with two interceding TMHs, and either one (BceB) or two periplasmic domains (YbbP; Figure 8). As previously noted by Milton Saier, one permutation that appears to be absent in this ABC superfamily is the fusion of two permease domains and two NBDs into a single polypeptide (Khwaja et al., 2005). In cases where the TMDs represent an apparent fusion of two monomers, the NBD is always encoded as a separate protein (Khwaja et al., 2005).

## Conclusions and future directions

MacB and its homologs confer resistance to antibiotics, but the current evidence suggests they do not transport substrates across the cytoplasmic membrane. Crystal and cryo-EM structures demonstrate how the distinctive MacB architecture uses a mechanotransmission mechanism in which the energy from cytoplasmic ATP hydrolysis is communicated through transmembrane movements to perform useful work in the extracytoplasmic space. Despite recent advances, many questions remain. The absence of a substrate-bound structure is one of the biggest barriers to our collective understanding of MacB-type ABC transporters. Substrate-bound structures of other ABC exporters are the exception, not the rule (Johnson and Chen, 2017; Mi et al., 2017), but they would demonstrate how substrate is recognized by the MacB periplasmic domain. The N-terminus of MacA affects the ATPase activity of MacB but this region has not been resolved in any of the current structures; clearly structural information demonstrating how this region of MacA can affect MacB is vital to completely understand the mechanism.

All the available structures have been determined using detergent-solubilised proteins but the presence of a membrane environment dramatically alters the ATPase activity of MacB and its response to MacA (Tikhonova et al., 2007; Picard et al., 2018). Mass spectrometry analysis of purified MacB suggested it specifically binds phosphatidylethanolamine molecules (Barrera et al., 2009) while different phospholipids were found to differentially affect the activity of the MacB homolog LolCDE (Miyamoto and Tokuda, 2007). Structures of MacB within the context of a lipid bilayer may help reveal how specific phospholipids can modulate transporter activity.

Another important line of enquiry will be to better understand the range of substrates that MacB-like proteins interact with. The peptide substrates of MacB and its homologs are commonly small disulfide bonded peptides reminiscent of some antimicrobial peptides such as mammalian defensins. Expression of MacB-like proteins influences survival of *Salmonella* and *Streptococcus pyogenes* in macrophages (Phelps and Neely, 2007; Bogomolnaya et al., 2013). Whether MacB can protect pathogenic bacteria within host systems by efflux of defensins or other substrates bears further investigation. Moreover, the sheer structural diversity and size-range of the MacB substrate repertoire (ranging from ~0.5 kDa macrolides to 10 kDa proteins such as dispersin) requires explanation.

In *Bacillus*, an additional membrane protein that is not found in conventional tripartite systems from Gram-negative bacteria, YknW, associates with and affects the activity of the Gram-positive MacAB homolog YknXYZ. Furthermore, a small membrane protein, AcrZ, modulates the action of the *E. coli* AcrAB-TolC TEP (Hobbs et al., 2012). Many small membrane proteins in *E. coli* remain uncharacterized (Storz et al., 2014), and so the possibility of other proteins affecting the activity of MacB cannot be excluded.

MacB mediated antibiotic resistance, and the involvement of its homologs in different facets of bacterial physiology make Type VII ABC transporters an attractive target for antibiotic therapy. Inhibitors of MacAB have not been isolated, but screening of chemical libraries identified two different inhibitors that target the MacB homolog LolCDE (McLeod et al., 2015; Nayar et al., 2015; Nickerson et al., 2018). Mutations conferring resistance map to the stalk helices, and interestingly, one of these inhibitors stimulated the ATPase activity of LolCDE *in vitro* (Nickerson et al., 2018). These inhibitors may therefore function by uncoupling ATP hydrolysis from the mechanotransmissive movement of the extracytoplasmic domains. Clearly, a general class of inhibitors that could interrupt mechanotransmission would be immediately useful as tools to study MacB-like transporters and, in the longer term, as potential new antibiotics. Indeed, targeting MacB-like ABC transporters for inhibition is particularly attractive, not only because FtsEX and LolCDE are essential, but also because this class of transporters is absent in humans.

The importance of the MacB architecture in bacterial physiology is becoming increasingly apparent. The recent structures now provide a template to understand its action not only in antibiotic resistance but in underpinning a variety of fundamental bacterial cell processes which are themselves targets for antimicrobial therapy.

## Author contributions

NG wrote the draft manuscript. AC and EK prepared the figures. All authors read and revised the final manuscript.

### Conflict of interest statement

The authors declare that the research was conducted in the absence of any commercial or financial relationships that could be construed as a potential conflict of interest. The reviewer AN declared a shared affiliation, with no collaboration, with several of the authors, NG, EK, VK, to the handling Editor.

## References

[B1] Aires J. R. Nikaido H. (2005). Aminoglycosides are captured from both periplasm and cytoplasm by the AcrD multidrug efflux transporter of *Escherichia coli*. J. Bacteriol. 187, 1923–1929. 10.1128/JB.187.6.1923-1929.2005 15743938 PMC1064028

[B2] Akama H. Kanemaki M. Yoshimura M. Tsukihara T. Kashiwagi T. Yoneyama H.. (2004). Crystal structure of the drug discharge outer membrane protein, OprM, of *Pseudomonas aeruginosa*: dual modes of membrane anchoring and occluded cavity end. J. Biol. Chem. 279, 52816–52819. 10.1074/jbc.C400445200 15507433

[B3] Al-Hamad A. Upton M. Burnie J. (2009). Molecular cloning and characterization of SmrA, a novel ABC multidrug efflux pump from *Stenotrophomonas maltophilia*. J. Antimicrob. Chemother. 64, 731–734. 10.1093/jac/dkp271 19643774

[B4] Aller S. G. Yu J. Ward A. Weng Y. Chittaboina S. Zhuo R.. (2009). Structure of P-glycoprotein reveals a molecular basis for poly-specific drug binding. Science 323, 1718–1722. 10.1126/science.1168750 19325113 PMC2720052

[B5] Bader M. W. Sanowar S. Daley M. E. Schneider A. R. Cho U. Xu W.. (2005). Recognition of antimicrobial peptides by a bacterial sensor kinase. Cell 122, 461–472. 10.1016/j.cell.2005.05.030 16096064

[B6] Balibar C. J. Vaillancourt F. H. Walsh C. T. (2005). Generation of D amino acid residues in assembly of arthrofactin by dual condensation/epimerization domains. Chem. Biol. 12, 1189–1200. 10.1016/j.chembiol.2005.08.010 16298298

[B7] Barrera N. P. Isaacson S. C. Zhou M. Bavro V. N. Welch A. Schaedler T. A.. (2009). Mass spectrometry of membrane transporters reveals subunit stoichiometry and interactions. Nat. Methods 6, 585–587. 10.1038/nmeth.1347 19578383 PMC4066579

[B8] Bavro V. N. Pietras Z. Furnham N. Pérez-Cano L. Fernández-Recio J. Pei X. Y.. (2008). Assembly and channel opening in a bacterial drug efflux machine. Mol. Cell 30, 114–121. 10.1016/j.molcel.2008.02.015 18406332 PMC2292822

[B9] Bibb L. A. Schmitt M. P. (2010). The ABC transporter HrtAB confers resistance to hemin toxicity and is regulated in a hemin-dependent manner by the ChrAS two-component system in *Corynebacterium diphtheriae*. J. Bacteriol. 192, 4606–4617. 10.1128/JB.00525-10 20639324 PMC2937406

[B10] Bogomolnaya L. M. Andrews K. D. Talamantes M. Maple A. Ragoza Y. Vazquez-Torres A.. (2013). The ABC-type efflux pump MacAB protects *Salmonella enterica* serovar typhimurium from oxidative stress. MBio 4, e00630–e00613. 10.1128/mBio.00630-13 24169575 PMC3809562

[B11] Bountra K. Hagelueken G. Choudhury H. G. Corradi V. El Omari K. Wagner A.. (2017). Structural basis for antibacterial peptide self-immunity by the bacterial ABC transporter McjD. EMBO J. 36, 3062–3079. 10.15252/embj.201797278 28864543 PMC5641919

[B12] Cho H. Kang H. (2012). The PseEF efflux system is a virulence factor of *Pseudomonas syringae* pv. syringae. J. Microbiol. 50, 79–90. 10.1007/s12275-012-1353-9 22367941

[B13] Choudhury H. G. Tong Z. Mathavan I. Li Y. Iwata S. Zirah S.. (2014). Structure of an antibacterial peptide ATP-binding cassette transporter in a novel outward occluded state. Proc. Natl. Acad. Sci. U.S.A. 111, 9145–9150. 10.1073/pnas.1320506111 24920594 PMC4078857

[B14] Collins B. Curtis N. Cotter P. D. Hill C. Ross R. P. (2010). The ABC transporter AnrAB contributes to the innate resistance of Listeria monocytogenes to nisin, bacitracin, and various beta-lactam antibiotics. Antimicrob. Agents Chemother. 54, 4416–4423. 10.1128/AAC.00503-10 20643901 PMC2944581

[B15] Coumes-Florens S. Brochier-Armanet C. Guiseppi A. Denizot F. Foglino M. (2011). A new highly conserved antibiotic sensing/resistance pathway in firmicutes involves an ABC transporter interplaying with a signal transduction system. PLoS ONE 6:e15951. 10.1371/journal.pone.0015951 21283517 PMC3023708

[B16] Crow A. Greene N. P. Kaplan E. Koronakis V. (2017). Structure and mechanotransmission mechanism of the MacB ABC transporter superfamily. Proc. Natl. Acad. Sci. U.S.A. 114, 12572–12577. 10.1073/pnas.1712153114 29109272 PMC5703307

[B17] Daury L. Orange F. Taveau J.-C. Verchère A. Monlezun L. Gounou C.. (2016). Tripartite assembly of RND multidrug efflux pumps. Nat. Commun. 7:10731. 10.1038/ncomms10731 26867482 PMC4754349

[B18] Dawson R. J. Locher K. P. (2006). Structure of a bacterial multidrug ABC transporter. Nature 443, 180–185. 10.1038/nature05155 16943773

[B19] Diaz M. Valdivia E. Martínez-Bueno M. Fernández M. Soler-González A. S. Ramírez-Rodrigo H.. (2003). Characterization of a new operon, as-48EFGH, from the as-48 gene cluster involved in immunity to enterocin AS-48. Appl. Environ. Microbiol. 69, 1229–1236. 10.1128/AEM.69.2.1229-1236.2003 12571051 PMC143590

[B20] Dintner S. Heermann R. Fang C. Jung K. Gebhard S. (2014). A sensory complex consisting of an ATP-binding cassette transporter and a two-component regulatory system controls bacitracin resistance in *Bacillus subtilis*. J. Biol. Chem. 289, 27899–27910. 10.1074/jbc.M114.596221 25118291 PMC4183823

[B21] Dintner S. Staron A. Berchtold E. Petri T. Mascher T. Gebhard S. (2011). Coevolution of ABC transporters and two-component regulatory systems as resistance modules against antimicrobial peptides in Firmicutes Bacteria. J. Bacteriol. 193, 3851–3862. 10.1128/JB.05175-11 21665979 PMC3147537

[B22] Draper L. A. Cotter P. D. Hill C. Ross R. P. (2015). Lantibiotic resistance. Microbiol. Mol. Biol. Rev. 79, 171–191. 10.1128/MMBR.00051-14 25787977 PMC4394878

[B23] Dubern J.-F. Coppoolse E. R. Stiekema W. J. Bloemberg G. V. (2008). Genetic and functional characterization of the gene cluster directing the biosynthesis of putisolvin I and II in *Pseudomonas putida* strain PCL1445. Microbiology 154, 2070–2083. 10.1099/mic.0.2008/016444-0 18599835

[B24] Du D. Wang Z. James N. R. Voss J. E. Klimont E. Ohene-Agyei T.. (2014). Structure of the AcrAB-TolC multidrug efflux pump. Nature 509, 512–515. 10.1038/nature13205 24747401 PMC4361902

[B25] Du S. Pichoff S. Lutkenhaus J. (2016). FtsEX acts on FtsA to regulate divisome assembly and activity. Proc. Natl. Acad. Sci. U.S.A. 113, E5052–E5061. 10.1073/pnas.1606656113 27503875 PMC5003251

[B26] Eicher T. Cha H. Seeger M. A. Brandstätter L. El-Delik J. Bohnert J. A.. (2012). Transport of drugs by the multidrug transporter AcrB involves an access and a deep binding pocket that are separated by a switch-loop. Proc. Natl. Acad. Sci. U.S.A. 109, 5687–5692. 10.1073/pnas.1114944109 22451937 PMC3326505

[B27] Federici L. Du D. Walas F. Matsumura H. Fernandez-Recio J. McKeegan K. S.. (2005). The crystal structure of the outer membrane protein VceC from the bacterial pathogen *Vibrio cholerae* at 1.8 A resolution. J. Biol. Chem. 280, 15307–15314. 10.1074/jbc.M500401200 15684414

[B28] Fitzpatrick A. W. P. Llabrés S. Neuberger A. Blaza J. N. Bai X.-C. Okada U.. (2017). Structure of the MacAB–TolC ABC-type tripartite multidrug efflux pump. Nat. Microbiol. 2:17070. 10.1038/nmicrobiol.2017.70 28504659 PMC5447821

[B29] Foreman D. T. Martinez Y. Coombs G. Torres A. Kupersztoch Y. M. (1995). ToIC and DsbA are needed for the secretion of STB, a heat-stable enterotoxin of *Escherichia coli*. Mol. Microbiol. 18, 237–245. 10.1111/j.1365-2958.1995.mmi_18020237.x 8709843

[B30] Garti-Levi S. Hazan R. Kain J. Fujita M. Ben-Yehuda S. (2008). The FtsEX ABC transporter directs cellular differentiation in *Bacillus subtilis*. Mol. Microbiol. 69, 1018–1028. 10.1111/j.1365-2958.2008.06340.x 18573177

[B31] González C. Langdon G. M. Bruix M. Gálvez A. Valdivia E. Maqueda M.. (2000). Bacteriocin AS-48, a microbial cyclic polypeptide structurally and functionally related to mammalian NK-lysin. Proc. Natl. Acad. Sci. U.S.A. 97, 11221–11226. 10.1073/pnas.210301097 11005847 PMC17181

[B32] Greene N. P. Hinchliffe P. Crow A. Ababou A. Hughes C. Koronakis V. (2013). Structure of an atypical periplasmic adaptor from a multidrug efflux pump of the spirochete *Borrelia burgdorferi*. FEBS Lett. 587, 2984–2988. 10.1016/j.febslet.2013.06.056 23851070 PMC3807786

[B33] Guan H.-H. Yoshimura M. Chuankhayan P. Lin C.-C. Chen N.-C. Yang M.-C.. (2015). Crystal structure of an antigenic outer-membrane protein from *Salmonella Typhi* suggests a potential antigenic loop and an efflux mechanism. Sci. Rep. 5:16441. 10.1038/srep16441 26563565 PMC4643347

[B34] Hannauer M. Schäfer M. Hoegy F. Gizzi P. Wehrung P. Mislin G. L. A.. (2012). Biosynthesis of the pyoverdine siderophore of *Pseudomonas aeruginosa* involves precursors with a myristic or a myristoleic acid chain. FEBS Lett. 586, 96–101. 10.1016/j.febslet.2011.12.004 22172280

[B35] Hannauer M. Yeterian E. Martin L. W. Lamont I. L. Schalk I. J. (2010). An efflux pump is involved in secretion of newly synthesized siderophore by *Pseudomonas aeruginosa*. FEBS Lett. 584, 4751–4755. 10.1016/j.febslet.2010.10.051 21035449

[B36] Harley K. T. Djordjevic G. M. Tseng T.-T. Saier M. H. (2000). Membrane-fusion protein homologues in Gram-positive bacteria. Mol. Microbiol. 36, 516–517. 10.1046/j.1365-2958.2000.01866.x 10792737

[B37] Henry R. Vithanage N. Harrison P. Seemann T. Coutts S. Moffatt J. H.. (2012). Colistin-resistant, lipopolysaccharide-deficient *Acinetobacter baumannii* responds to lipopolysaccharide loss through increased expression of genes involved in the synthesis and transport of lipoproteins, phospholipids, and poly-β-1,6-N-acetylglucos. Antimicrob. Agents Chemother. 56, 59–69. 10.1128/AAC.05191-11 22024825 PMC3256090

[B38] Higgins M. K. Bokma E. Koronakis E. Hughes C. Koronakis V. (2004). Structure of the periplasmic component of a bacterial drug efflux pump. Proc. Natl. Acad. Sci. U.S.A. 101, 9994–9999. 10.1073/pnas.0400375101 15226509 PMC454203

[B39] Hinchliffe P. Greene N. P. Paterson N. G. Crow A. Hughes C. Koronakis V. (2014). Structure of the periplasmic adaptor protein from a major facilitator superfamily (MFS) multidrug efflux pump. FEBS Lett. 588, 3147–3153. 10.1016/j.febslet.2014.06.055 24996185 PMC4158417

[B40] Hinchliffe P. Symmons M. F. Hughes C. Koronakis V. (2013). Structure and operation of bacterial tripartite pumps. Annu. Rev. Microbiol. 67, 221–242. 10.1146/annurev-micro-092412-155718 23808339

[B41] Hobbs E. C. Yin X. Paul B. J. Astarita J. L. Storz G. (2012). Conserved small protein associates with the multidrug efflux pump AcrB and differentially affects antibiotic resistance. Proc. Natl. Acad. Sci. U.S.A. 109, 16696–16701. 10.1073/pnas.1210093109 23010927 PMC3478662

[B42] Hollenstein K. Frei D. C. Locher K. P. (2007). Structure of an ABC transporter in complex with its binding protein. Nature 446, 213–216. 10.1038/nature05626 17322901

[B43] Imperi F. Tiburzi F. Visca P. (2009). Molecular basis of pyoverdine siderophore recycling in *Pseudomonas aeruginosa*. Proc. Natl. Acad. Sci. U.S.A. 106, 20440–20445. 10.1073/pnas.0908760106 19906986 PMC2787144

[B44] Johnson Z. L. Chen J. (2017). Structural basis of substrate recognition by the multidrug resistance protein MRP1. Cell 168, 1075.e9–1085.e9. 10.1016/j.cell.2017.01.041 28238471

[B45] Johnson Z. L. Chen J. (2018). ATP binding enables substrate release from multidrug resistance protein 1. Cell 172, 81.e10–83.e10. 10.1016/j.cell.2017.12.005 29290467

[B46] Joubert L. Derré-Bobillot A. Gaudu P. Gruss A. Lechardeur D. (2014). HrtBA and menaquinones control haem homeostasis in *Lactococcus lactis*. Mol. Microbiol. 93, 823–833. 10.1111/mmi.12705 25040434

[B47] Kallenberg F. Dintner S. Schmitz R. Gebhard S. (2013). Identification of regions important for resistance and signalling within the antimicrobial peptide transporter BceAB of *Bacillus subtilis*. J. Bacteriol. 195, 3287–3297. 10.1128/JB.00419-13 23687272 PMC3697649

[B48] Khwaja M. Ma Q. Saier M. H. (2005). Topological analysis of integral membrane constituents of prokaryotic ABC efflux systems. Res. Microbiol. 156, 270–277. 10.1016/j.resmic.2004.07.010 15748994

[B49] Kingston A. W. Zhao H. Cook G. M. Helmann J. D. (2014). Accumulation of heptaprenyl diphosphate sensitizes *Bacillus subtilis* to bacitracin: implications for the mechanism of resistance mediated by the BceAB transporter. Mol. Microbiol. 93, 37–49. 10.1111/mmi.12637 24806199 PMC4077933

[B50] Khosa S. AlKhatib Z. Smits S. H. J. (2013). NSR from Streptococcus agalactiae confers resistance against nisin and is encoded by a conserved nsr operon. Biol. Chem. 394, 1543–1549. 10.1515/hsz-2013-0167 23893686

[B51] Kobayashi N. Nishino K. Hirata T. Yamaguchi A. (2003). Membrane topology of ABC-type macrolide antibiotic exporter MacB in *Escherichia coli*. FEBS Lett. 546, 241–246. 10.1016/S0014-5793(03)00579-9 12832048

[B52] Kobayashi N. Nishino K. Yamaguchi A. (2001). Novel macrolide-specific ABC-type efflux transporter in *Escherichia coli*. J. Bacteriol. 183, 5639–5644. 10.1128/JB.183.19.5639-5644.2001 11544226 PMC95455

[B53] Korkhov V. M. Mireku S. A. Locher K. P. (2012). Structure of AMP-PNP-bound vitamin B12 transporter BtuCD–F. Nature 490, 367–372. 10.1038/nature11442 23000901

[B54] Koronakis V. Sharff A. Koronakis E. Luisi B. Hughes C. (2000). Crystal structure of the bacterial membrane protein TolC central to multidrug efflux and protein export. Nature 405, 914–919. 10.1038/35016007 10879525

[B55] Kulathila R. Kulathila R. Indic M. van den Berg B. (2011). Crystal structure of *Escherichia coli* CusC, the outer membrane component of a heavy metal efflux pump. PLoS ONE 6:e15610. 10.1371/journal.pone.0015610 21249122 PMC3017539

[B56] Lee J.-Y. Kinch L. N. Borek D. M. Wang J. Wang J. Urbatsch I. L.. (2016). Crystal structure of the human sterol transporter ABCG5/ABCG8. Nature 533, 561–564. 10.1038/nature17666 27144356 PMC4964963

[B57] Lim S. P. Roongsawang N. Washio K. Morikawa M. (2009). Flexible exportation mechanisms of arthrofactin in *Pseudomonas* sp. MIS38. J. Appl. Microbiol. 107, 157–166. 10.1111/j.1365-2672.2009.04189.x 19302333

[B58] Lin D. Y. Huang S. Chen J. (2015a). Crystal structures of a polypeptide processing and secretion transporter. Nature 523, 425–430. 10.1038/nature14623 26201595

[B59] Lin H. T. Bavro V. N. Barrera N. P. Frankish H. M. Velamakanni S. Van Veen H. W.. (2009). MacB ABC transporter is a dimer whose ATPase activity and macrolide-binding capacity are regulated by the membrane fusion protein MacA. J. Biol. Chem. 284, 1145–1154. 10.1074/jbc.M806964200 18955484 PMC2613632

[B60] Lin H.-T. V. Massam-Wu T. Lin C.-P. Wang Y.-J. A. Shen Y.-C. Lu W.-J.. (2017). The *Vibrio cholerae* var regulon encodes a metallo-β-lactamase and an antibiotic efflux pump, which are regulated by VarR, a LysR-type transcription factor. PLoS ONE 12:e0184255. 10.1371/journal.pone.0184255 28898293 PMC5595328

[B61] Lin M.-F. Lin Y.-Y. Tu C.-C. Lan C.-Y. (2015b). Distribution of different efflux pump genes in clinical isolates of multidrug-resistant *Acinetobacter baumannii* and their correlation with antimicrobial resistance. J. Microbiol. Immunol. Infect. 50, 224–231. 10.1016/j.jmii.2015.04.004 26055688

[B62] Lin Y. T. Huang Y. W. Liou R. S. Chang Y. C. Yang T. C. (2014). MacABCsm, an ABC-type tripartite efflux pump of *Stenotrophomonas maltophilia* involved in drug resistance, oxidative and envelope stress tolerances and biofilm formation. J. Antimicrob. Chemother. 69, 3221–3226. 10.1093/jac/dku317 25139838

[B63] Liu W.-T. Yang Y.-L. Xu Y. Lamsa A. Haste N. M. Yang J. Y.. (2010). Imaging mass spectrometry of intraspecies metabolic exchange revealed the cannibalistic factors of *Bacillus subtilis*. Proc. Natl. Acad. Sci. U.S.A. 107, 16286–16290. 10.1073/pnas.1008368107 20805502 PMC2941286

[B64] Li W. Rokni-Zadeh H. De Vleeschouwer M. Ghequire M. G. K. Sinnaeve D. Xie G.-L.. (2013). The antimicrobial compound xantholysin defines a new group of *Pseudomonas* cyclic lipopeptides. PLoS ONE 8:e62946. 10.1371/journal.pone.0062946 23690965 PMC3656897

[B65] Locher K. P. (2016). Mechanistic diversity in ATP-binding cassette (ABC) transporters. Nat. Struct. Mol. Biol. 23, 487–493. 10.1038/nsmb.3216 27273632

[B66] Lomovskaya O. Lewis K. (1992). Emr, an *Escherichia coli* locus for multidrug resistance. Proc. Natl. Acad. Sci. U.S.A. 89, 8938–8942. 10.1073/pnas.89.19.8938 1409590 PMC50039

[B67] Lubelski J. Konings W. N. Driessen A. J. M. (2007). Distribution and physiology of ABC-Type transporters contributing to multidrug resistance in bacteria. Microbiol. Mol. Biol. Rev. 71, 463–476. 10.1128/MMBR.00001-07 17804667 PMC2168643

[B68] Lu S. Zgurskaya H. I. (2012). Role of ATP binding and hydrolysis in assembly of MacAB-TolC macrolide transporter. Mol. Microbiol. 86, 1132–1143. 10.1111/mmi.12046 23057817 PMC3508387

[B69] Lu S. Zgurskaya H. I. (2013). MacA, a periplasmic membrane fusion protein of the macrolide transporter MacAB-TolC, binds lipopolysaccharide core specifically and with high affinity. J. Bacteriol. 195, 4865–4872. 10.1128/JB.00756-13 23974027 PMC3807484

[B70] Luo Q. Yang X. Yu S. Shi H. Wang K. Xiao L.. (2017). Structural basis for lipopolysaccharide extraction by ABC transporter LptB2FG. Nat. Struct. Mol. Biol. 24, 469–474. 10.1038/nsmb.3399 28394325

[B71] Majchrzykiewicz J. A. Kuipers O. P. Bijlsma J. J. E. (2010). Generic and specific adaptive responses of *Streptococcus pneumoniae* to challenge with three distinct antimicrobial peptides, bacitracin, LL-37, and nisin. Antimicrob. Agents Chemother. 54, 440–451. 10.1128/AAC.00769-09 19917758 PMC2798553

[B72] Matias V. R. Beveridge T. J. (2006). Native cell wall organization shown by cryo-electron microscopy confirms the existence of a periplasmic space in *Staphylococcus aureus*. J. Bacteriol. 188, 1011–1021. 10.1128/JB.188.3.1011-1021.2006 16428405 PMC1347357

[B73] Matson J. S. Livny J. DiRita V. J. (2017). A putative *Vibrio cholerae* two-component system controls a conserved periplasmic protein in response to the antimicrobial peptide polymyxin B. PLoS ONE 12:e0186199. 10.1371/journal.pone.0186199 29020117 PMC5636140

[B74] Matsuo T. Chen J. Minato Y. Ogawa W. Mizushima T. Kuroda T.. (2008). SmdAB, a heterodimeric ABC-Type multidrug efflux pump, in *Serratia marcescens*. J. Bacteriol. 190, 648–654. 10.1128/JB.01513-07 18024518 PMC2223691

[B75] Mavrici D. Marakalala M. J. Holton J. M. Prigozhin D. M. Gee C. L. Zhang Y. J.. (2014). *Mycobacterium tuberculosis* FtsX extracellular domain activates the peptidoglycan hydrolase, RipC. Proc. Natl. Acad. Sci. U.S.A. 111, 8037–8042. 10.1073/pnas.1321812111 24843173 PMC4050617

[B76] McLeod S. M. Fleming P. R. MacCormack K. McLaughlin R. E. Whiteaker J. D. Narita S.-I.. (2015). Small-molecule inhibitors of gram-negative lipoprotein trafficking discovered by phenotypic screening. J. Bacteriol. 197, 1075–1082. 10.1128/JB.02352-14 25583975 PMC4336340

[B77] Meehl M. Herbert S. Götz F. Cheung A. (2007). Interaction of the GraRS two-component system with the VraFG ABC transporter to support vancomycin-intermediate resistance in Staphylococcus aureus. Antimicrob. Agents Chemother. 51, 2679–2689. 10.1128/AAC.00209-07 17502406 PMC1932546

[B78] Mikolosko J. Bobyk K. Zgurskaya H. I. Ghosh P. (2006). Conformational flexibility in the multidrug efflux system protein AcrA. Structure 14, 577–587. 10.1016/j.str.2005.11.015 16531241 PMC1997295

[B79] Mi W. Li Y. Yoon S. H. Ernst R. K. Walz T. Liao M. (2017). Structural basis of MsbA-mediated lipopolysaccharide transport. Nature 549, 233–237. 10.1038/nature23649 28869968 PMC5759761

[B80] Miyamoto S. Tokuda H. (2007). Diverse effects of phospholipids on lipoprotein sorting and ATP hydrolysis by the ABC transporter LolCDE complex. Biochim. Biophys. Acta 1768, 1848–1854. 10.1016/j.bbamem.2007.04.005 17498646

[B81] Modali S. D. Zgurskaya H. I. (2011). The periplasmic membrane proximal domain of MacA acts as a switch in stimulation of ATP hydrolysis by MacB transporter. Mol. Microbiol. 81, 937–951. 10.1111/j.1365-2958.2011.07744.x 21696464 PMC3177148

[B82] Murakami S. Nakashima R. Yamashita E. Matsumoto T. Yamaguchi A. (2006). Crystal structures of a multidrug transporter reveal a functionally rotating mechanism. Nature 443, 173–179. 10.1038/nature05076 16915237

[B83] Murakami S. Nakashima R. Yamashita E. Yamaguchi A. (2002). Crystal structure of bacterial multidrug efflux transporter AcrB. Nature 419, 587–593. 10.1038/nature01050 12374972

[B84] Nakashima R. Sakurai K. Yamasaki S. Nishino K. Yamaguchi A. (2011). Structures of the multidrug exporter AcrB reveal a proximal multisite drug-binding pocket. Nature 480, 565–569. 10.1038/nature10641 22121023

[B85] Narita S. I. Tokuda H. (2017). Bacterial lipoproteins; biogenesis, sorting and quality control. Biochim. Biophys. Acta 1862, 1414–1423. 10.1016/j.bbalip.2016.11.009 27871940

[B86] Nayar A. S. Dougherty T. J. Ferguson K. E. Granger B. A. McWilliams L. Stacey C.. (2015). Novel antibacterial targets and compounds revealed by a high-throughput cell wall reporter assay. J. Bacteriol. 197, 1726–1734. 10.1128/JB.02552-14 25733621 PMC4402386

[B87] Nickerson N. N. Jao C. C. Xu Y. Quinn J. Skippington E. Alexander M. K.. (2018). A novel inhibitor of the LolCDE ABC transporter essential for lipoprotein trafficking in Gram-negative bacteria. Antimicrob. Agents Chemother. 62:e02151-17. 10.1128/AAC.02151-17 29339384 PMC5913989

[B88] Nishi J. Sheikh J. Mizuguchi K. Luisi B. Burland V. Boutin A.. (2003). The export of coat protein from enteroaggregative *Escherichia coli* by a specific ATP-binding cassette transporter system. J. Biol. Chem. 278, 45680–45689. 10.1074/jbc.M306413200 12933818

[B89] Nishino K. Latifi T. Groisman E. A. (2006). Virulence and drug resistance roles of multidrug efflux systems of *Salmonella enterica* serovar Typhimurium. Mol. Microbiol. 59, 126–141. 10.1111/j.1365-2958.2005.04940.x 16359323

[B90] Okada U. Yamashita E. Neuberger A. Morimoto M. van Veen H. W. Murakami S. (2017). Crystal structure of tripartite-type ABC transporter MacB from *Acinetobacter baumannii*. Nat. Commun. 8:1336. 10.1038/s41467-017-01399-2 29109439 PMC5673888

[B91] Pei X.-Y. Hinchliffe P. Symmons M. F. Koronakis E. Benz R. Hughes C.. (2011). Structures of sequential open states in a symmetrical opening transition of the TolC exit duct. Proc. Natl. Acad. Sci. U.S.A. 108, 2112–2117. 10.1073/pnas.1012588108 21245342 PMC3033246

[B92] Perez C. Gerber S. Boilevin J. Bucher M. Darbre T. Aebi M.. (2015). Structure and mechanism of an active lipid-linked oligosaccharide flippase. Nature 524, 433–438. 10.1038/nature14953 26266984

[B93] Phelps H. A. Neely M. N. (2007). SalY of the *Streptococcus pyogenes* lantibiotic locus is required for full virulence and intracellular survival in macrophages. Infect. Immun. 75, 4541–4551. 10.1128/IAI.00518-07 17576754 PMC1951192

[B94] Picard M. Tikhonova E. B. Broutin I. Lu S. Verchère A. Zgurskaya H. I. (2018). Biochemical reconstitution and characterization of multicomponent drug efflux transporters Methods Mol. Biol. 1700, 113–145. 10.1007/978-1-4939-7454-2_8 29177829

[B95] Pilonieta M. C. Bodero M. D. Munson G. P. (2007). CfaD-dependent expression of a novel extracytoplasmic protein from enterotoxigenic *Escherichia coli*. J. Bacteriol. 189, 5060–5067. 10.1128/JB.00131-07 17496090 PMC1951884

[B96] Popella P. Krauss S. Ebner P. Nega M. Deibert J. Götz F. (2016). VraH Is the Third component of the *Staphylococcus aureus* VraDEH system involved in gallidermin and daptomycin resistance and pathogenicity. Antimicrob. Agents Chemother. 60, 2391–2401. 10.1128/AAC.02865-15 26856834 PMC4808217

[B97] Prost L. R. Sanowar S. Miller S. I. (2007). Salmonella sensing of anti-microbial mechanisms to promote survival within macrophages. Immunol. Rev. 219, 55–65. 10.1111/j.1600-065X.2007.00557.x 17850481

[B98] Quigley N. B. Mo Y.-Y. Gross D. C. (1993). SyrD is required for syringomycin production by *Pseudomonas syringae* pathovar syringae and is related to a family of ATP-binding secretion proteins. Mol. Microbiol. 9, 787–801. 10.1111/j.1365-2958.1993.tb01738.x 8231810

[B99] Quistgaard E. M. Löw C. Guettou F. Nordlund P. (2016). Understanding transport by the major facilitator superfamily (MFS): structures pave the way. Nat. Rev. Mol. Cell Biol. 17, 123–132. 10.1038/nrm.2015.25 26758938

[B100] Ramachandra M. Ambudkar S. V. Chen D. Hrycyna C. A. Dey S. Gottesman M. M.. (1998). Human P-Glycoprotein exhibits reduced affinity for substrates during a catalytic transition state ^†^. Biochemistry 37, 5010–5019. 10.1021/bi973045u 9538020

[B101] Reuter G. Janvilisri T. Venter H. Shahi S. Balakrishnan L. van Veen H. W. (2003). The ATP binding cassette multidrug transporter LmrA and lipid transporter MsbA have overlapping substrate specificities. J. Biol. Chem. 278, 35193–35198. 10.1074/jbc.M306226200 12842882

[B102] Rietkötter E. Hoyer D. Mascher T. (2008). Bacitracin sensing in *Bacillus subtilis*. Mol. Microbiol. 68, 768–785. 10.1111/j.1365-2958.2008.06194.x 18394148

[B103] Rouquette-Loughlin C. E. Balthazar J. T. Shafer W. M. (2005). Characterization of the MacA-MacB efflux system in Neisseria gonorrhoeae. J. Antimicrob. Chemother. 56, 856–860. 10.1093/jac/dki333 16162665

[B104] Schmidt K. L. Peterson N. D. Kustusch R. J. Wissel M. C. Graham B. Phillips G. J.. (2004). A predicted ABC transporter, FtsEX, is needed for cell division in *Escherichia coli*. J. Bacteriol. 186, 785–793. 10.1128/JB.186.3.785-793.2004 14729705 PMC321481

[B105] Seeger M. A. Schiefner A. Eicher T. Verrey F. Diederichs K. Pos K. M. (2006). Structural asymmetry of AcrB trimer suggests a peristaltic pump mechanism. Science 313, 1295–1298. 10.1126/science.1131542 16946072

[B106] Sham L.-T. Barendt S. M. Kopecky K. E. Winkler M. E. (2011). Essential PcsB putative peptidoglycan hydrolase interacts with the essential FtsXSpn cell division protein in *Streptococcus pneumoniae* D39. Proc. Natl. Acad. Sci. U.S.A. 108, E1061–E1069. 10.1073/pnas.1108323108 22006325 PMC3215045

[B107] Singh H. Velamakanni S. Deery M. J. Howard J. Wei S. L. van Veen H. W. (2016). ATP-dependent substrate transport by the ABC transporter MsbA is proton-coupled. Nat. Commun. 7:12387. 10.1038/ncomms12387 27499013 PMC4979069

[B108] Staron P. Forchhammer K. Maldener I. (2011). Novel ATP-driven pathway of glycolipid export involving TolC protein. J. Biol. Chem. 286, 38202–38210. 10.1074/jbc.M111.269332 21917923 PMC3207437

[B109] Staron P. Forchhammer K. Maldener I. (2014). Structure-function analysis of the ATP-driven glycolipid efflux pump DevBCA reveals complex organization with TolC/HgdD. FEBS Lett. 588, 395–400. 10.1016/j.febslet.2013.12.004 24361095

[B110] Stauff D. L. Bagaley D. Torres V. J. Joyce R. Anderson K. L. Kuechenmeister L.. (2008). *Staphylococcus aureus* HrtA Is an ATPase required for protection against heme toxicity and prevention of a transcriptional heme stress response. J. Bacteriol. 190, 3588–3596. 10.1128/JB.01921-07 18326576 PMC2395006

[B111] Storz G. Wolf Y. I. Ramamurthi K. S. (2014). Small proteins can no longer be ignored. Annu. Rev. Biochem. 83, 753–777. 10.1146/annurev-biochem-070611-102400 24606146 PMC4166647

[B112] Su C.-C. Radhakrishnan A. Kumar N. Long F. Bolla J. R. Lei H.-T.. (2014). Crystal structure of the *Campylobacter jejuni* CmeC outer membrane channel. Protein Sci. 23, 954–961. 10.1002/pro.2478 24753291 PMC4088979

[B113] Tal N. Schuldiner S. (2009). A coordinated network of transporters with overlapping specificities provides a robust survival strategy. Proc. Natl. Acad. Sci. U.S.A. 106, 9051–9056. 10.1073/pnas.0902400106 19451626 PMC2690002

[B114] Tanabe M. Szakonyi G. Brown K. A. Henderson P. J. F. Nield J. Byrne B. (2009). The multidrug resistance efflux complex, EmrAB from *Escherichia coli* forms a dimer *in vitro*. Biochem. Biophys. Res. Commun. 380, 338–342. 10.1016/j.bbrc.2009.01.081 19171121

[B115] ter Beek J. Guskov A. Slotboom D. J. (2014). Structural diversity of ABC transporters. J. Gen. Physiol. 143, 419–435. 10.1085/jgp.201411164 24638992 PMC3971661

[B116] Thanabalu T. Koronakis E. Hughes C. Koronakis V. (1998). Substrate-induced assembly of a contiguous channel for protein export from *E. coli*: reversible bridging of an inner-membrane translocase to an outer membrane exit pore. EMBO J. 17, 6487–6496. 9822594 10.1093/emboj/17.22.6487 PMC1170996

[B117] Tikhonova E. B. Devroy V. K. Lau S. Y. Zgurskaya H. I. (2007). Reconstitution of the *Escherichia coli* macrolide transporter: the periplasmic membrane fusion protein MacA stimulates the ATPase activity of MacB. Mol. Microbiol. 63, 895–910. 10.1111/j.1365-2958.2006.05549.x 17214741

[B118] Tsuda H. Yamashita Y. Shibata Y. Nakano Y. Koga T. (2002). Genes involved in bacitracin resistance in Streptococcus mutans. Antimicrob. Agents Chemother. 46, 3756–3764. 10.1128/AAC.46.12.3756-3764.2002 12435673 PMC132740

[B119] Turlin E. Heuck G. Simões Brandão M. I. Szili N. Mellin J. R. Lange N.. (2014). Protoporphyrin (PPIX) efflux by the MacAB-TolC pump in *Escherichia coli*. Microbiologyopen 3, 849–859. 10.1002/mbo3.203 25257218 PMC4263509

[B120] van Veen H. W. Venema K. Bolhuis H. Oussenko I. Kok J. Poolman B.. (1996). Multidrug resistance mediated by a bacterial homolog of the human multidrug transporter MDR1. Proc. Natl. Acad. Sci. U.S.A. 93, 10668–10672. 10.1073/pnas.93.20.10668 8855237 PMC38212

[B121] Velarde J. J. Varney K. M. Inman K. G. Farfan M. Dudley E. Fletcher J.. (2007). Solution structure of the novel dispersin protein of enteroaggregative *Escherichia coli*. Mol. Microbiol. 66, 1123–1135. 10.1111/j.1365-2958.2007.05985.x 17986189

[B122] Wakeman C. A. Hammer N. D. Stauff D. L. Attia A. S. Anzaldi L. L. Dikalov S. I.. (2012). Menaquinone biosynthesis potentiates haem toxicity in *Staphylococcus aureus*. Mol. Microbiol. 86, 1376–1392. 10.1111/mmi.12063 23043465 PMC3524387

[B123] Wang B. Dukarevich M. Sun E. I. Yen M. R. Saier M. H. (2009). Membrane porters of ATP-Binding cassette transport systems are polyphyletic. J. Membr. Biol. 231, 1–10. 10.1007/s00232-009-9200-6 19806386 PMC2760711

[B124] Wang Z. Fan G. Hryc C. F. Blaza J. N. Serysheva I. I. Schmid M. F.. (2017). An allosteric transport mechanism for the AcrAB-TolC multidrug efflux pump. Elife 6, 1–19. 10.7554/eLife.24905 28355133 PMC5404916

[B125] Woebking B. Reuter G. Shilling R. A. Velamakanni S. Shahi S. Venter H.. (2005). Drug-lipid A interactions on the *Escherichia coli* ABC transporter MsbA. J. Bacteriol. 187, 6363–6369. 10.1128/JB.187.18.6363-6369.2005 16159769 PMC1236644

[B126] Xu K. Zhang M. Zhao Q. Yu F. Guo H. Wang C.. (2013). Crystal structure of a folate energy-coupling factor transporter from *Lactobacillus brevis*. Nature 497, 268–271. 10.1038/nature12046 23584589

[B127] Xu Y. Guo J. Wang L. Jiang R. Jin X. Liu J.. (2016). The crystal structure of the YknZ extracellular domain of ABC transporter YknWXYZ from *Bacillus amyloliquefaciens*. PLoS ONE 11:e0155846. 10.1371/journal.pone.0155846 27243566 PMC4887032

[B128] Xu Y. Jo I. Wang L. Chen J. Fan S. Dong Y.. (2017). Hexameric assembly of membrane fusion protein YknX of the sporulation delaying efflux pump from *Bacillus amyloliquefaciens*. Biochem. Biophys. Res. Commun. 493, 152–157. 10.1016/j.bbrc.2017.09.059 28917834

[B129] Xu Y. Moeller A. Jun S.-Y. Le M. Yoon B.-Y. Kim J.-S.. (2012). Assembly and channel opening of outer membrane protein in tripartite drug efflux pumps of Gram-negative bacteria. J. Biol. Chem. 287, 11740–11750. 10.1074/jbc.M111.329375 22308040 PMC3320922

[B130] Xu Y. Sim S. H. Ki H. N. Xiao L. J. Kim H. M. Kwang Y. H.. (2009). Crystal structure of the periplasmic region of MacB, a noncanonic ABC transporter. Biochemistry 48, 5218–5225. 10.1021/bi900415t 19432486

[B131] Xu Y. Sim S. H. Song S. Piao S. Kim H. M. Jin X. L.. (2010). The tip region of the MacA alpha-hairpin is important for the binding to TolC to the *Escherichia coli* MacAB-TolC pump. Biochem. Biophys. Res. Commun. 394, 962–965. 10.1016/j.bbrc.2010.03.097 20307498

[B132] Xu Y. Song S. Moeller A. Kim N. Piao S. Sim S. H.. (2011). Functional implications of an intermeshing cogwheel-like interaction between TolC and MacA in the action of macrolide-specific efflux pump MacAB-TolC. J. Biol. Chem. 286, 13541–13549. 10.1074/jbc.M110.202598 21325274 PMC3075700

[B133] Yakushi T. Masuda K. Narita S. Matsuyama S. Tokuda H. (2000). A new ABC transporter mediating the detachment of lipid-modified proteins from membranes. Nat. Cell Biol. 2, 212–218. 10.1038/35008635 10783239

[B134] Yamada Y. Tikhonova E. B. Zgurskaya H. I. (2012). YknWXYZ is an unusual four-component transporter with a role in protection against sporulation-delaying-protein-induced killing of *Bacillus subtilis*. J. Bacteriol. 194, 4386–4394. 10.1128/JB.00223-12 22707703 PMC3416220

[B135] Yamanaka H. Kobayashi H. Takahashi E. Okamoto K. (2008). MacAB is involved in the secretion of *Escherichia coli* heat-stable enterotoxin II. J. Bacteriol. 190, 7693–7698. 10.1128/JB.00853-08 18805970 PMC2583606

[B136] Yang D. C. Peters N. T. Parzych K. R. Uehara T. Markovski M. Bernhardt T. G. (2011). An ATP-binding cassette transporter-like complex governs cell-wall hydrolysis at the bacterial cytokinetic ring. Proc. Natl. Acad. Sci. U.S.A. 108, E1052–E1060. 10.1073/pnas.1107780108 22006326 PMC3215046

[B137] Yang H.-B. Hou W.-T. Cheng M.-T. Jiang Y.-L. Chen Y. Zhou C.-Z. (2018). Structure of a MacAB-like efflux pump from *Streptococcus pneumoniae*. Nat. Commun. 9:196. 10.1038/s41467-017-02741-4 29335499 PMC5768738

[B138] Yonehara R. Yamashita E. Nakagawa A. (2016). Crystal structures of OprN and OprJ, outer membrane factors of multidrug tripartite efflux pumps of *Pseudomonas aeruginosa*. Proteins 84, 759–769. 10.1002/prot.25022 26914226

[B139] Yum S. Xu Y. Piao S. Sim S. H. Kim H. M. Jo W. S.. (2009). Crystal structure of the periplasmic component of a tripartite macrolide-specific efflux pump. J. Mol. Biol. 387, 1286–1297. 10.1016/j.jmb.2009.02.048 19254725

[B140] Zgurskaya H. I. Yamada Y. Tikhonova E. B. Ge Q. Krishnamoorthy G. (2009). Structural and functional diversity of bacterial membrane fusion proteins. Biochim. Biophys. Acta 1794, 794–807. 10.1016/j.bbapap.2008.10.010 19041958

[B141] Zuber B. Haenni M. Ribeiro T. Minnig K. Lopes F. Moreillon P.. (2006). Granular layer in the periplasmic space of Gram-positive bacteria and fine structures of Enterococcus gallinarum and Streptococcus gordonii septa revealed by cryo-electron microscopy of vitreous sections. J. Bacteriol. 188, 6652–6660. 10.1128/JB.00391-06 16952957 PMC1595480

[B142] Zutz A. Hoffmann J. Hellmich U. A. Glaubitz C. Ludwig B. Brutschy B.. (2011). Asymmetric ATP hydrolysis cycle of the heterodimeric multidrug ABC transport complex TmrAB from thermus thermophilus. J. Biol. Chem. 286, 7104–7115. 10.1074/jbc.M110.201178 21190941 PMC3044967

[B143] Zwama M. Yamasaki S. Nakashima R. Sakurai K. Nishino K. Yamaguchi A. (2018). Multiple entry pathways within the efflux transporter AcrB contribute to multidrug recognition. Nat. Commun. 9:124. 10.1038/s41467-017-02493-1 29317622 PMC5760665

