# Corrigendum: Antibiotic Resistance Mediated by the MacB ABC Transporter Family: A Structural and Functional Perspective

**DOI:** 10.3389/fmicb.2018.02318

**Published:** 2018-09-28

**Authors:** Nicholas P. Greene, Elise Kaplan, Allister Crow, Vassilis Koronakis

**Affiliations:** ^1^Department of Pathology, University of Cambridge, Cambridge, United Kingdom; ^2^School of Life Sciences, University of Warwick, Coventry, United Kingdom

**Keywords:** antibiotic resistance, tripartite efflux pump, MacB, mechanotransmission, ABC transporter, lantibiotic, membrane protein, antimicrobial resistance

In the original article, there was a mistake in Figure 6 as published. The labels “ATP hydrolysis” and “ATP binding” were inverted; the corrected Figure 6 appears below. The error does not change the scientific conclusions of the article in any way.

**Figure 6:**
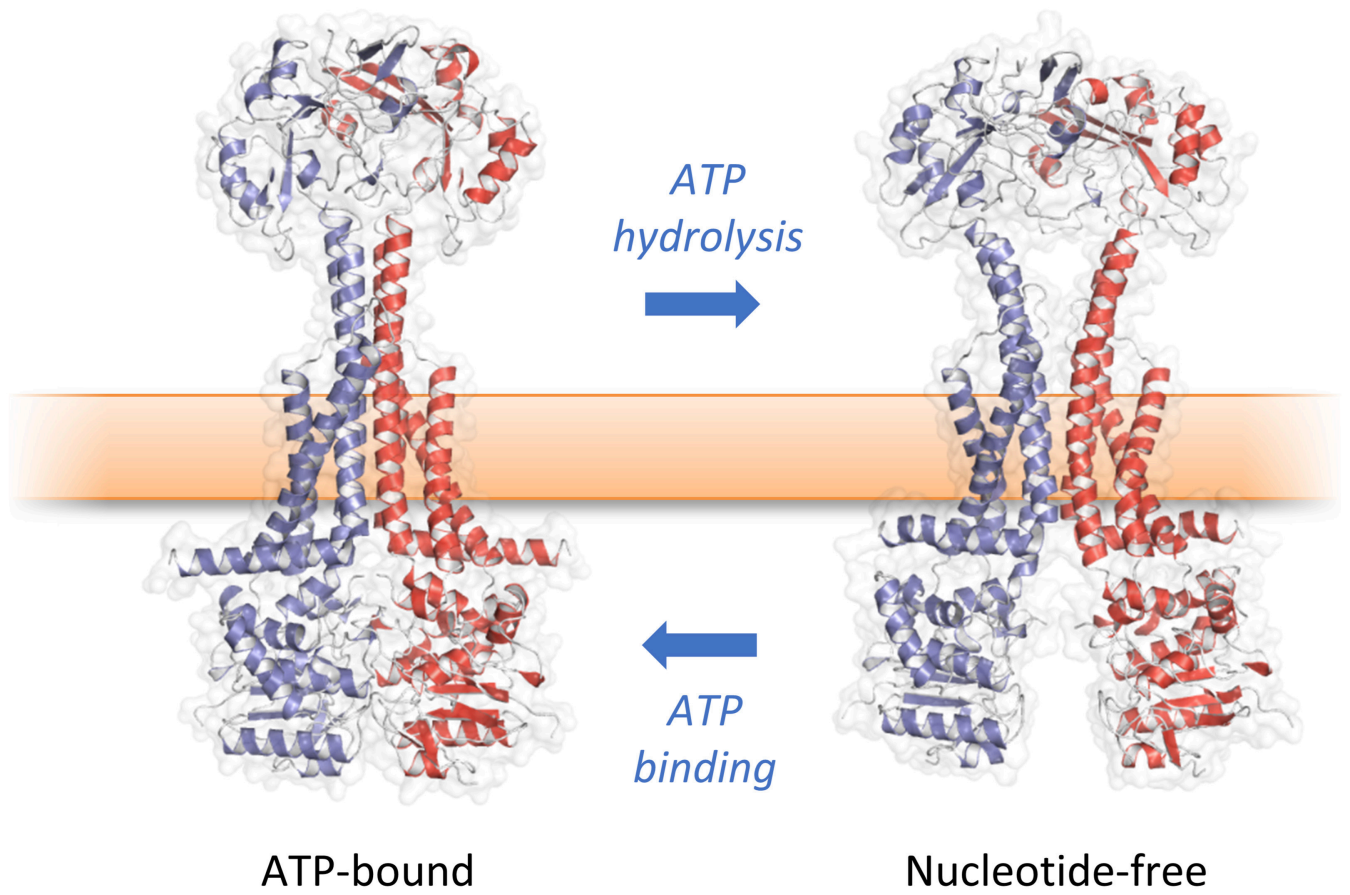
Mechanotransmission mechanism of MacB. ATP binding and hydrolysis cause large, transmembrane conformational changes in MacB structure. Rather than transporting substrates across the inner membrane, MacB-like proteins coordinate reversible dimerization of their NBDs with periplasmic conformational changes. TEP-forming MacB homologs use periplasmic conformational change to drive substrates across the bacterial outer membrane via TolC-like exit ducts. MacB homologs that do not form TEPs are proposed to use similar motions during lipoprotein trafficking and transmembrane signaling. Adapted from Crow et al. (2017).

The original article has been updated.

## Conflict of interest statement

The authors declare that the research was conducted in the absence of any commercial or financial relationships that could be construed as a potential conflict of interest.
